# Incorporating mesopelagic fish into the evaluation of conservation areas for marine living resources under climate change scenarios

**DOI:** 10.1007/s42995-023-00188-9

**Published:** 2023-08-15

**Authors:** Shuhao Liu, Yang Liu, Katharina Teschke, Mark A. Hindell, Rachel Downey, Briannyn Woods, Bin Kang, Shuyang Ma, Chi Zhang, Jianchao Li, Zhenjiang Ye, Peng Sun, Jianfeng He, Yongjun Tian

**Affiliations:** 1 Research Centre for Deep Sea and Polar Fisheries, and Key Laboratory of Mariculture, Ministry of Education, Ocean University of China, Qingdao, 266003 China; 2 Frontiers Science Center for Deep Ocean Multispheres and Earth System, Ocean University of China, Qingdao, 266100 China; 3 Alfred Wegener Institute, Helmholtz Centre for Polar and Marine Research, Am Handelshafen 12, 27570 Bremerhaven, Germany; 4 Helmholtz Institute for Functional Marine Biodiversity at the University Oldenburg, Ammerländer Heerstraße 231, 23129 Oldenburg, Germany; 5 Institute for Marine and Antarctic Studies, University of Tasmania, Hobart, 7004 Australia; 6 Fenner School of Environment and Society, Australian National University, Canberra, ACT 2602 Australia; 7 College of Fisheries, Ocean University of China, Qingdao, 266003 China; 8 Polar Research Institute of China, Shanghai, 200136 China

**Keywords:** Myctophids, Mesopelagic fish, Species distribution model, Southern Ocean, Antarctic Peninsula

## Abstract

Mesopelagic fish (meso-fish) are central species within the Southern Ocean (SO). However, their ecosystem role and adaptive capacity to climate change are rarely integrated into protected areas assessments. This is a pity given their importance as crucial prey and predators in food webs, coupled with the impacts of climate change. Here, we estimate the habitat distribution of nine meso-fish using an ensemble model approach (MAXENT, random forest, and boosted regression tree). Four climate model simulations were used to project their distribution under two representative concentration pathways (RCP4.5 and RCP8.5) for short-term (2006–2055) and long-term (2050–2099) periods. In addition, we assess the ecological representativeness of protected areas under climate change scenarios using meso-fish as indicator species. Our models show that all species shift poleward in the future. Lanternfishes (family Myctophidae) are predicted to migrate poleward more than other families (Paralepididae, Nototheniidae, Bathylagidae, and Gonostomatidae). In comparison, lanternfishes were projected to increase habitat area in the eastern SO but lose area in the western SO; the opposite was projected for species in other families. Important areas (IAs) of meso-fish are mainly distributed near the Antarctic Peninsula and East Antarctica. Negotiated protected area cover 23% of IAs at present and 38% of IAs in the future (RCP8.5, long-term future). Many IAs of meso-fish still need to be included in protected areas, such as the Prydz Bay and the seas around the Antarctic Peninsula. Our results provide a framework for evaluating protected areas incorporating climate change adaptation strategies for protected areas management.

**Supplementary Information:**

The online version contains supplementary material available at 10.1007/s42995-023-00188-9.

## Introduction

Mesopelagic fishes (meso-fish) play a significant role in the regional food webs in the Southern Ocean (SO) and carbon cycle between lower and upper trophic levels, surface waters, and the deep ocean (Collins et al. 2012; Davison et al. 2015; Woods et al. 2022, 2023). Meso-fish inhabit the open ocean’s twilight zone (200–1000 m) and dominate the total fish biomass in the world’s oceans with more than 1000 million tonnes (Irigoien et al. 2014). In the Southern Ocean (SO), meso-fish are central in the transfer of energy (Woods et al. 2023). They are key prey for higher predators (e.g., penguins, seals, and seabirds), and they are also the primary consumers of secondary producers (e.g., copepods and euphausiids) (McCormack et al. 2021; Saunders et al. 2015a, 2019). A significant proportion of meso-fish exhibit diel vertical migration (Gjøsaeter and Kawaguchi 1980) and are thus a significant conduit of carbon transport from the surface to the deep sea (Saba et al. 2021). Antarctic krill (*Euphausia superba*) is a crucial species in the SO food web. Meso-fish are also critical predators of krill, and link primary producers to higher predators through both krill-dependent and krill-independent trophic pathways (Saunders et al. 2015a, 2019). Therefore, meso-fish are recognized to be dominant in alternative trophic pathways that can equal or even exceed the importance of the krill pathway in some areas, or when krill are scarce (McCormack et al. 2020, 2021).

Although the ecological importance of meso-fish is widely recognized, they remain the least studied ecosystem components (Dowd et al. 2022; Woods et al. 2022). Over preceding decades, an increasing number of studies has been conducted to model the spatial distribution of Antarctic species, mainly for higher predators (e.g., seals, seabirds and penguins) (Hindell et al. 2020), Antarctic krill (Sylvester et al. 2021), benthic organisms, e.g., sea urchins and sea stars (Charlène et al. 2020; Fabri-Ruiz et al. 2020), cephalopods (Xavier et al. 2016), and copepods (Pinkerton et al. 2010). However, relatively little research has been done on meso-fish biomass, life history, and special distribution under environmental and climate change scenarios (Dowd et al. 2022; Duhamel et al. 2014; Freer et al. 2019; Kaartvedt et al. 2012; Loots et al. 2007; Ran et al. 2022). This is partly due to the ability of meso-fish to evade and escape from sampling gears (Collins et al. 2012; Davison et al. 2015; Kaartvedt et al. 2012) and also a general lack of commercial interest from fisheries (Caiger et al. 2021). These reasons have limited our knowledge of the spatiotemporal variability of meso-fish and processes that environmental predictors shape their distribution, especially in the context of climate change.

Recent studies suggest that the meso-fish habitat in the SO would shift poleward under different climate change scenarios (Freer et al. 2019). Climate change is triggering marine fish responses, including a distribution shift as the change of different layers of environmental and biological components (Cheung et al. 2013). Climate change imposes pressure on Antarctic fish migration and recruitment in the SO (Caccavo et al. 2021). Moreover, these combined effects on meso-fish could in turn have implications for higher predators and low trophic levels, thus affecting the SO ecosystem (Caccavo et al. 2021; Freer et al. 2019; Saunders et al. 2019).

The rate and direction of meso-fish migration as the response to climate change may not be consistent across different SO regions. In particular, the SO is already showing a range of responses to climate change in the form of increasing ocean temperature and changes in sea ice concentration and extent (Constable et al. 2014; Gutt et al. 2015). However, only some parts of the SO have experienced an increase in oceanic temperature so far, whereas the Ross and Weddell Seas have experienced cooling and an increase in sea ice extent (Caccavo et al. 2021; Gutt et al. 2015; Trebilco et al. 2019). The rate and direction of changes in temperature and sea ice differ strongly among SO regions (Constable et al. 2014; Yang et al. 2021). The uneven effects of climate change across the SO demonstrate the complexity of the system. Hence, there is the need for further study of the meso-fish responses to climate change in different SO regions. Such knowledge will have important implications for conservation efforts, fisheries management, and the broader understanding of the impacts of climate change on marine ecosystems.

In climate change, marine protected areas (MPAs) are widely considered a mechanism for protecting species abundance under risks and biodiversity (Blowes et al. 2020; Gjerde et al. 2016; O’Regan et al. 2021). While MPAs cannot directly protect vulnerable species and their habitats from climate change, they can serve as refuges, where further anthropogenic impacts on the ecosystems are mitigated, providing species with a greater capacity to adapt to climate change (Roberts et al. 2017). Over the past 20 years, multiple calls have been made to establish scientifically based practical MPAs (Reisinger et al. 2022). In 2010, the Convention on Biological Diversity stipulated that an MPA network should protect at least 10% of marine areas by 2020. In 2014, the World Parks Congress increased the previous MPA objectives to at least 30% of protected marine areas by 2030 (O’Leary et al. 2016).

The Commission for the Conservation of Antarctic Marine Living Resources (CCAMLR) has implemented efforts to establish and propose MPAs to protect the SO. The importance of meso-fish and dynamic responses to climate change should be considered in the design of MPAs. In 2002, the CCAMLR officially recognized the World Summit on Sustainable Development’s commitment to establish a global MPA network, leading to the inclusion of MPAs as a standing agenda item (CCAMLR 2002). CCAMLR has since implemented two MPAs in the SO, the South Orkney Islands Southern Shelf MPA (SOISS MPA) (CCAMLR 2009), and the Ross Sea region MPA (RSr MPA) (CCAMLR 2016). Currently, 11.98% of the SO is covered by MPAs, and implementation of the MPAs currently being negotiated under CCAMLR (in East Antarctica, the Weddell Sea, and the area northwest of the Antarctic Peninsula) would protect 22% of the SO (Brooks et al. 2020). However, these MPAs are not enough; at least 30% coverage could be necessary to conserve biodiversity and maintain sustainable fisheries (Gaines et al. 2010; O’Leary et al. 2016; Roberts et al. 2020). Furthermore, the established and proposed MPAs under CCAMLR have primarily considered higher predators (seals, seabirds, penguins and whales), Antarctic toothfish, and Antarctic krill. In contrast, meso-fish have rarely been considered (CCAMLR 2002, 2009, 2016, 2020a, b). In addition, the CCAMLR MPAs have been designed based on present species distributions, and the dynamic responses of species to climate change have not been considered (Araújo et al. 2004; Fabri-Ruiz et al. 2020; Hindell et al. 2020).

In this study, we aimed to (1) explore the habitat distribution and species association of meso-fish with trophic connection and environmental changes; (2) project future meso-fish distribution and differences in habitat change between eastern and western regions of the SO; and (3) assess the rationality and ecological representativeness of protected areas under climate change scenarios using meso-fish as indicator species. Our ultimate goal of this study is to illustrate climate change's coupling effects on the special distribution of meso-fish in the SO. It provides a theoretical framework for policymakers to conserve the integrity of the SO ecosystem under cumulative impacts of climate change.

## Results

### The present-day distribution of mesopelagic fish

The true skill statistic (TSS) and area under the receiver operating characteristic curve (AUC) values showed high predictive performance for all three species distribution models (SDMs), random forest (RF), boosted regression tree (BRT), and maximum entropy (MAXENT) (Supplementary Figs. S3, S4). Seven environmental variables were used to construct SDMs: (i) sea ice area fraction (fractional coverage of a grid cell that is covered with sea ice), (ii) sea surface temperature (SST), (iii) sea surface salinity (SSS), (iv) temperature at 200 m (T_200), (v) salinity at 200 m (S_200), (vi) bathymetry (depth), and (vii) primary productivity (primary organic carbon production by all types of phytoplankton). The predictive performance among the three SDMs showed low variance (Supplementary Figs. S3, S4). All SDMs fitted with the environmental variables of the four Earth System Models (ESMs) had AUC values ranging from fair (0.7–0.8) to good (0.8–1.0), and TSS values ranging from fair (0.6–0.7) to good (0.7–0.9) (Freer et al. 2019). Averaged importance of environmental variables differed for the species (Supplementary Fig. S5). Among the lanternfish, *E. antarctica*, *K. anderssoni*, *G. braueri*, *G. nicholsi*, and *G. opisthopterus*, SST was the most important environmental variable, followed by T_200. The importance of SST and T_200 was higher than other environmental variables to a greater extent. For *N. coatsi*, the most crucial environmental variables were SST, followed by the sea ice area fraction. In contrast, the sea ice area fraction was the most important predictor for *P. antarctica*, *B. antarcticus*, and *C. microdon*. The importance of sea ice was higher than other environmental variables to a greater extent. The importance of environmental variables varied among the ESMs for most of the species. However, all four ESMs made a unanimous decision on identifying the most important environmental variables (Supplementary Fig. S6).

**Fig. 1:**
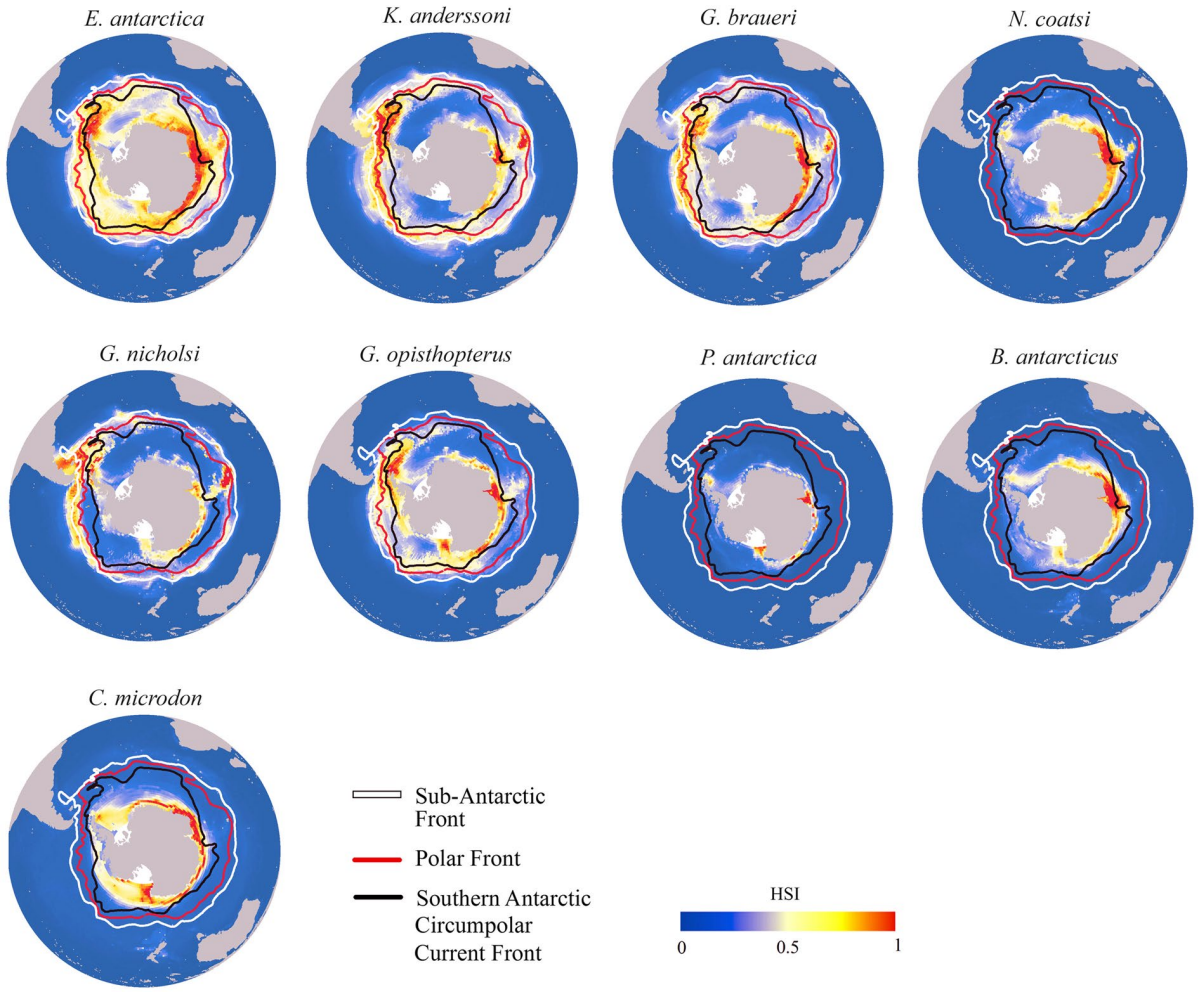
Present distribution of habitat suitability index (HSI) for nine mesopelagic fish species during the period of 1956–2005. The main oceanographic fronts are shown: the sub‐Antarctic Front (SAF; white line), Polar Front (PF; red line), and Southern Antarctic Circumpolar Current Front (SACCF; black line). The oceanographic front data were obtained from the Australian Antarctic Data Centre (Orsi & Harris 2019)

The ensemble model under the present-day (1956–2005) showed a circumpolar distribution for each mesopelagic species (Fig. 1). For the lanternfish, *E. antarctica*, *K. anderssoni*, *G. braueri*, *G. nicholsi*, *G. opisthopterus*, the present-day distribution ranged from the sub‐Antarctic Front (SAF) to the Antarctic continent, and these species were bound to the north by the SAF. *E. antarctica* had the most extensive distribution throughout the area from the SAF to the Antarctic continent. The core region of *E. antarctica* and *G. opisthopterus* species was distributed in the waters near the Antarctic Peninsula and the East Antarctic continent. This core region was distributed south of SACCF, and rarely extends north beyond SACCF. *K. anderssoni* and *G. braueri* were distributed between the SAF and SACCF, with the PF forming the center. *K. anderssoni* and *G. braueri* were predicted to have higher suitability along PF in western SO. In addition, the core region was distributed in the waters near the East Antarctic continent. Moreover, *G. nicholsi* showed a distribution from the PF to the SAF; this species was predicted to have higher suitability along SAF in western SO. All five lanternfish were predicted to have higher habitat suitability in areas of the continental shelf and slope in East Antarctica and near the Antarctic Peninsula, next to regions of the Ross Sea (e.g., *E. antarctica*, *G. opisthopterus*). Unlike lanternfish, *N. coatsi*, *P. antarctica*, *B. antarcticus*, and *C. microdon* (“high-Antarctic” species) were almost exclusively distributed on the Antarctic continental shelf and slope. For *C. microdon*, higher habitat suitability was predicted for nearly all circumpolar continental areas. In contrast, for the other “high-Antarctic” species, *N. coatsi*, *P. antarctica*, and *B. antarcticus*, high habitat suitability occurred only in relatively restricted East Antarctic and the Ross Sea regions.

**Fig. 2:**
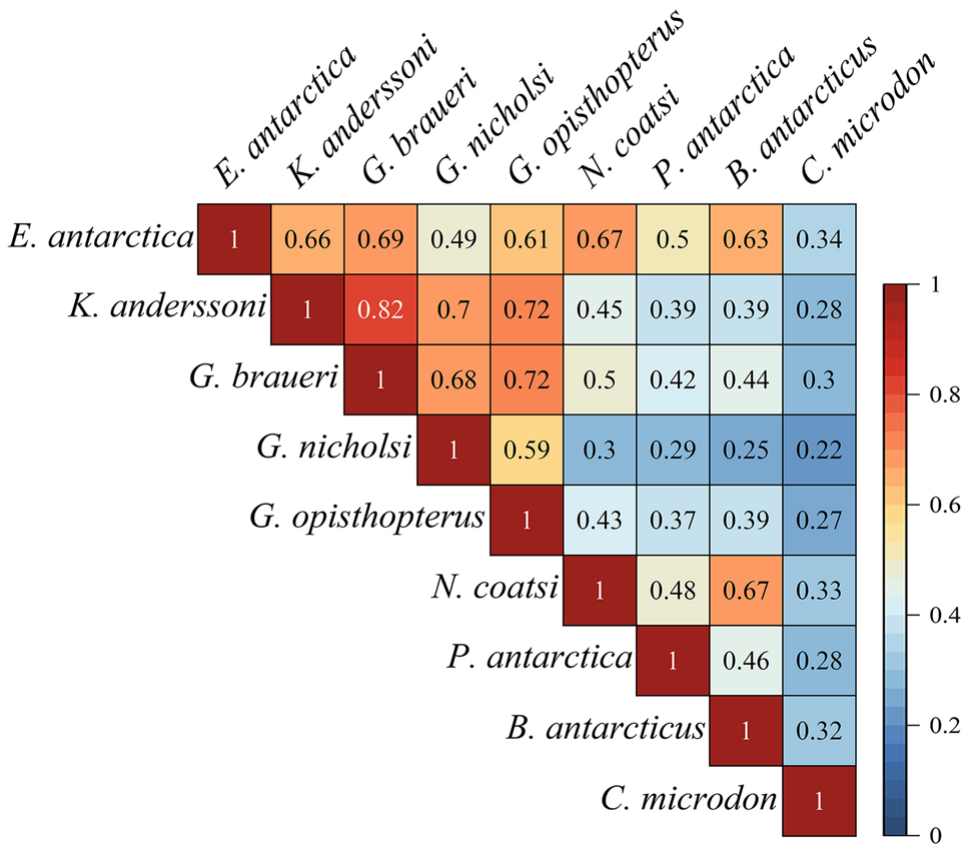
Averaged species associations were measured by residual correlation for nine mesopelagic fish species (99% highest posterior density interval)

All meso-fish had positive species associations (Fig. 2 and Supplementary Figs. S7–S10). The highest correlations were between the lanternfish, *E. antarctica*, *K. anderssoni*, *G. braueri*, *G. nicholsi*, and *G. opisthopterus*. Among lanternfish, *K. anderssoni* had the highest correlation (0.82) with *G. braueri*. *E. antarctica* had the lowest correlation (0.49) with *G. nicholsi*. *G. opisthopterus* had a high correlation (0.72) with *K. anderssoni* and *G. nicholsi.* There was a lower correlation between the lanternfish and “high-Antarctic” species (i.e., *N. coatsi, P. antarctica, B. antarcticus, C. microdon*). *G. nicholsi* showed the lowest correlation with *C. microdon* (0.22). Interestingly, *E. antarctica* showed relatively high correlations with all species except *C. microdon*. Without exception, this species has lower correlations with all meso-fish species. Among “high-Antarctic” species, there were only relatively low correlations to each other (except for *N. coatsi* with *B. antarcticus*). *C. microdon* had the lowest correlation (0.28) with *P. antarctica*.

### Short- and long-term future trends in habitat distribution

**Fig. 3:**
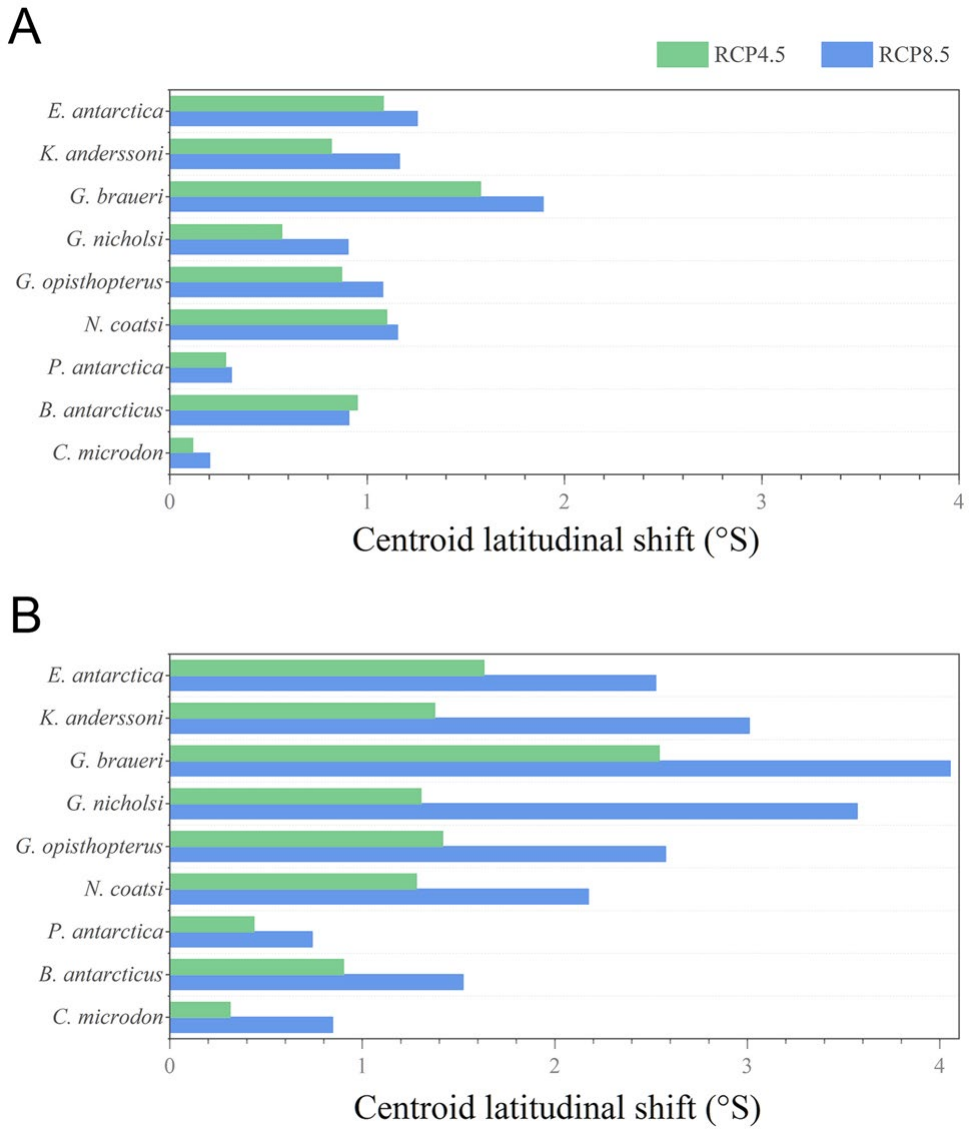
Change in suitable habitat in centroid latitudinal distribution for nine mesopelagic fish for the period (**A**) short-term future (2006–2055) and (**B**) long-term future (2050–2099) compared to the present-day (1956–2005), respectively, under RCP4.5 and RCP8.5. *RCP* Representative concentration pathway scenarios

For all meso-fish species, the centroid of suitable habitats was predicted to move poleward under RCP 4.5 (representative concentration pathway) and RCP8.5 in the short-term future (2006–2055) and long-term future (2050–2099), respectively (Fig. 3). All habitats migrated towards the pole, and a further poleward shift was shown in the long-term future (Fig. 3B) rather than the short-term future (Fig. 3A). For all species (except *B. antarcticus* in short-term future), a further poleward shift was identified under RCP8.5 than under RCP4.5. In the short-term future, four of the five greatest shifting meso-fish habitats were those of lanternfish. In the long-term future, the habitats of all five lanternfish exhibited more significant changes than “high-Antarctic” species under RCP4.5 and RCP8.5. The most significant shift in habitat was shown for *G. braueri* among all species for both periods and emission scenarios. Except for RCP8.5 in the long-term future, the habitat of *C. microdon* was predicted to move the least. The habitat of *N. coatsi* was predicted to shift the most among the “high-Antarctic” species for both periods and emission scenarios.

**Fig. 4:**
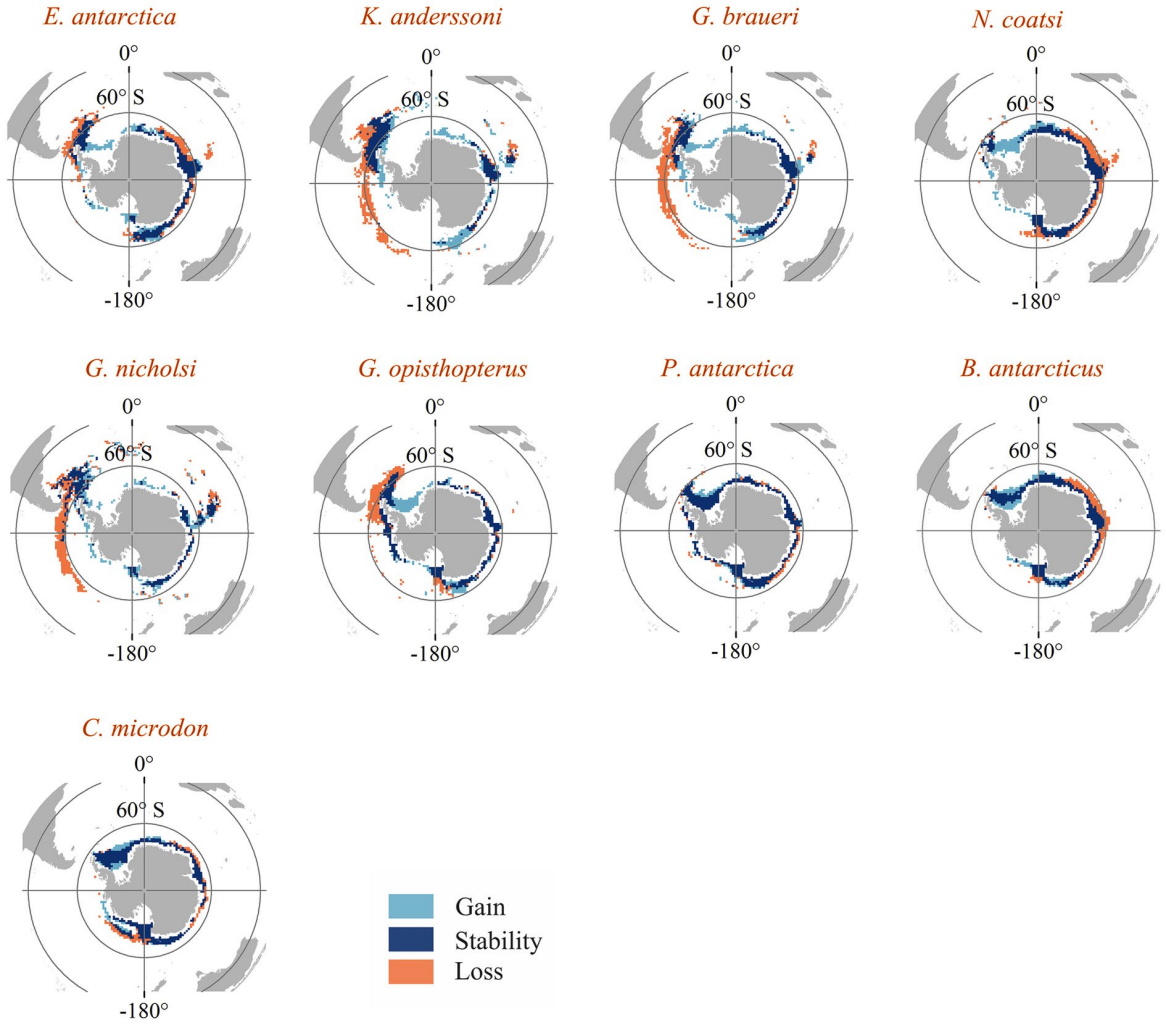
Change in suitable habitat in the predicted distribution for nine mesopelagic fish by long-term future (2050–2099) compared to the present-day (1956–2005) under the high emission scenario RCP8.5. The light blue, dark blue, and red areas represent habitat gain (habitat range expansion), habitat stability (no change in habitat), and habitat loss (habitat range contraction), respectively. *RCP* Representative concentration pathway scenarios

Suitable habitats of these species are all assessed to move under RCP8.5 in the long-term future (Fig. 4). The direction and severity of future variation in habitat distribution were different under each RCP and time period (Supplementary Figs. S11–S14). For each species, the suitable habitat would increase in poleward areas and decrease in northward areas in the short-term and long-term future under RCP4.5 and RCP8.5 relative to the present-day. The change of suitable habitat under RCP4.5 in the short-term and long-term future and RCP8.5 in the short-term had similar patterns (Supplementary Figs. S15–S17). For lanternfish, *E. antarctica*, *K. anderssoni*, *G. braueri*, *G. nicholsi*, and *G. opisthopterus*, the suitable habitats were lost in the areas around 60° S of the western SO and waters off the Antarctic Peninsula (Fig. 4). Suitable habitat gain for these species was predicted to occur in the Antarctic continental shelf and slope areas. For *K. anderssoni*, *G. braueri*, and *G. nicholsi*, the suitable habitat loss was almost along the 60° S in western SO. *E. antarctica* was also predicted to lose its suitable habitat in the East Antarctic continental shelf, and *G. opisthopterus* would lose suitable habitat in the Ross Sea. All lanternfish were predicted to lose suitable habitat in the lower latitudes waters around the Antarctic Peninsula and to stabilize suitable habitat in the higher latitudes around the Antarctic Peninsula. For *N. coatsi*, *P. antarctica*, *B. antarcticus*, and *C. microdon*, these species were predicted to lose suitable habitat in the shelf and slope areas of East Antarctica and expand suitable habitat in the high latitude waters of West Antarctica, especially in the Weddell Sea.

**Fig. 5:**
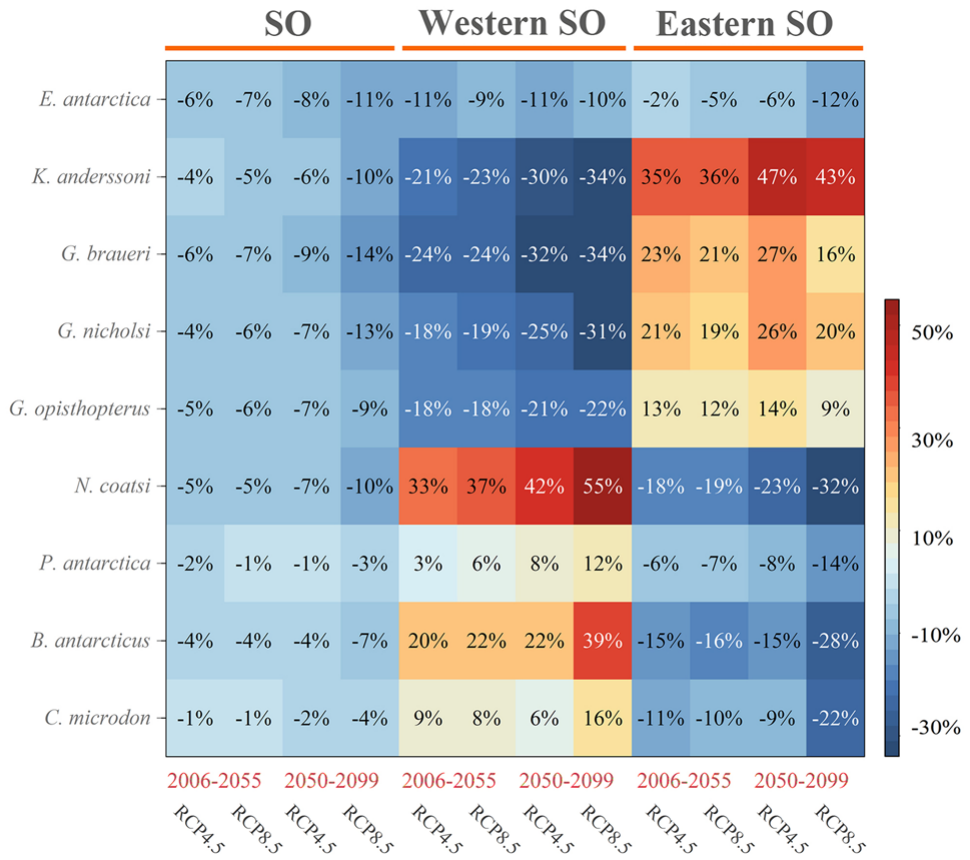
Percentage change in the area of suitable habitat for nine mesopelagic fish in the Southern Ocean (SO), western SO, and eastern SO by short-term future (2006–2055) and long-term future (2050–2099) compared to the present-day (1956–2005), respectively, under RCP4.5 and RCP8.5. *RCP* Representative concentration pathway scenarios

Suitable habitat for all meso-fish in the SO would decrease between 1% and 7% in the short-term future and between 1% and 14% in the long-term future under both climate scenarios compared to the present-day (Fig. 5). The five lanternfish species were predicted to experience a more significant loss of suitable habitat than the other species. The suitable habitat of *E. antarctica* would decrease in the western and eastern SO and more severely in western SO. The suitable habitat of lanternfish species (except *E. antarctica*) would reduce in the western SO and increase in the eastern SO. The suitable habitat of *K. anderssoni* and *G. braueri* was predicted to decrease by 34% each mostly in the western SO under RCP8.5 (long-term future). The suitable habitat of *K. anderssoni* would expand the most (47%) in the eastern SO under RCP4.5 (long-term future). For “high-Antarctic” species (i.e., *N. coatsi*, *P. antarctica*, *B. antarcticus*, *C. microdon*), suitable habitat was predicted to increase in the western SO and decrease in the eastern SO. The suitable habitat of *N. coatsi* would increase most in the western SO and decrease most in the eastern SO under RCP8.5 (long-term future) (+ 55% and – 32%, respectively). Overall, while all species were predicted to lose suitable habitat under climate change, there are differences in the eastern SO and western SO. Lanternfish in the open ocean tend to lose suitable habitat in the western SO, and gain in the eastern SO. In contrast, suitable habitats of other families in the neritic zone are likely to expand in the western SO, and shrink in the eastern SO. Overall, the suitable habitat loss was greater than the suitable habitat gain in the short- and long-term future under both climate scenarios.

### The overlap of meso-fish and krill with protected areas

**Fig. 6:**
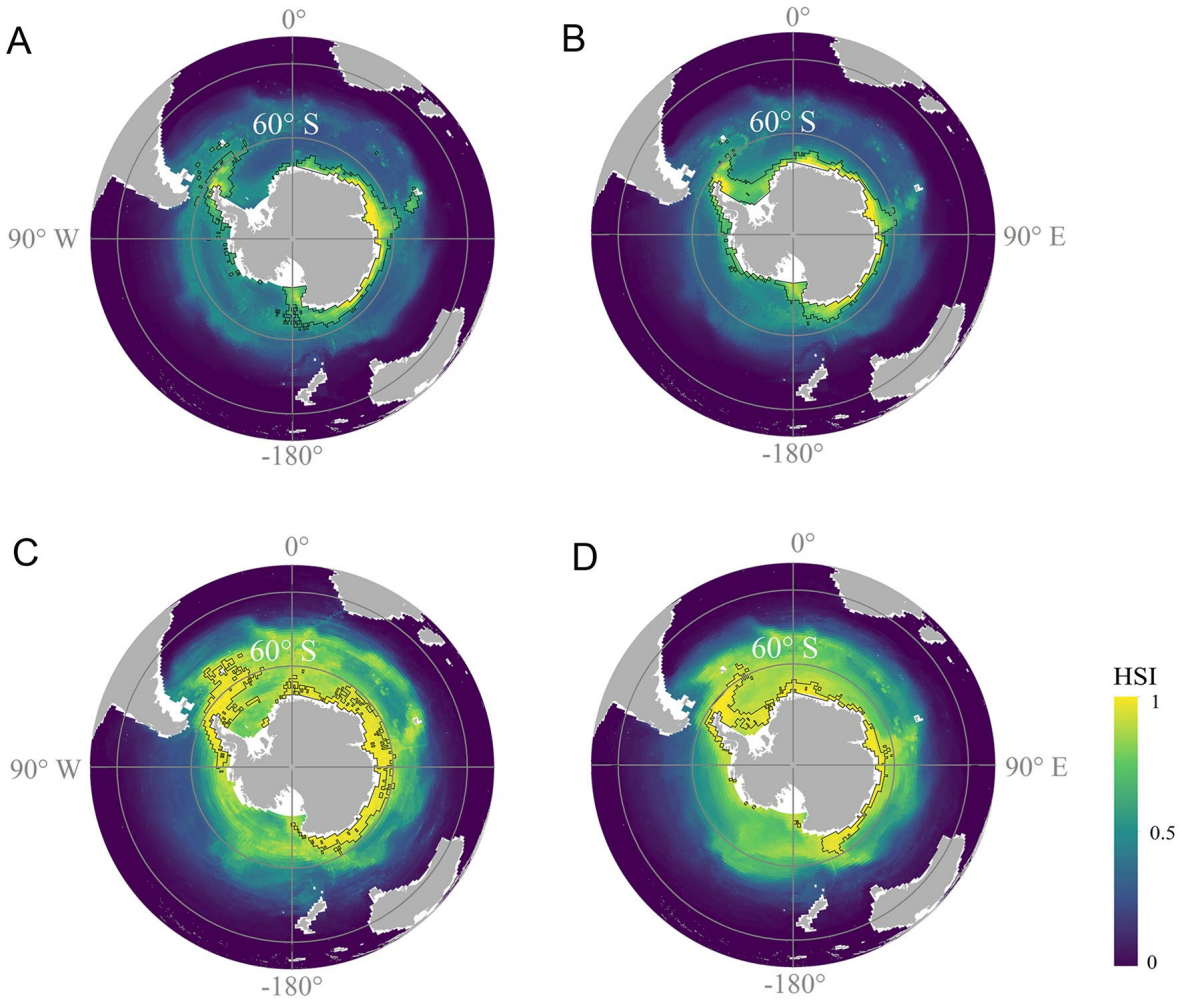
Distribution of mesopelagic fish under **A** present-day (1956–2005) and **B** RCP8.5 in the long-term future (2050–2099). The distribution of *E. superba* (krill) under **C** present-day (1956–2005) and **D** RCP8.5 in the long-term future (2050–2099). The areas within the black line represent important areas (IAs, top 5% HSI, habitat suitability index). *RCP* Representative concentration pathway scenarios

The spatial distribution of important areas (IAs) of meso-fish (Fig. 6A, B) and krill (Fig. 6C, D) showed that there were apparent differences between the present-day and future distribution patterns. Present-day IAs of meso-fish and krill were similarly distributed, with main occurrence north–northeast of the Antarctic Peninsula and on the East Antarctic continental shelf. Future IAs of meso-fish and krill were predicted to expand most in the Weddell Sea. In addition, the spatial expansion was predicted for meso-fish IAs in the Amundsen and Bellingshausen Seas in the long-term future under RCP8.5 (Fig. 6B). In the north–northeast region of the Antarctic Peninsula and the East Antarctic continental shelf, the IAs of meso-fish and krill would contract, and the IAs of meso-fish and krill would shrink to areas south of 60° S (Fig. 6B, D). The predictions of the RCP8.5 in the short-term future and of the RCP4.5 scenarios in the short-term and long-term future showed a similar spatial pattern in the IAs of meso-fish and krill (Supplementary Fig. S18).

**Fig. 7:**
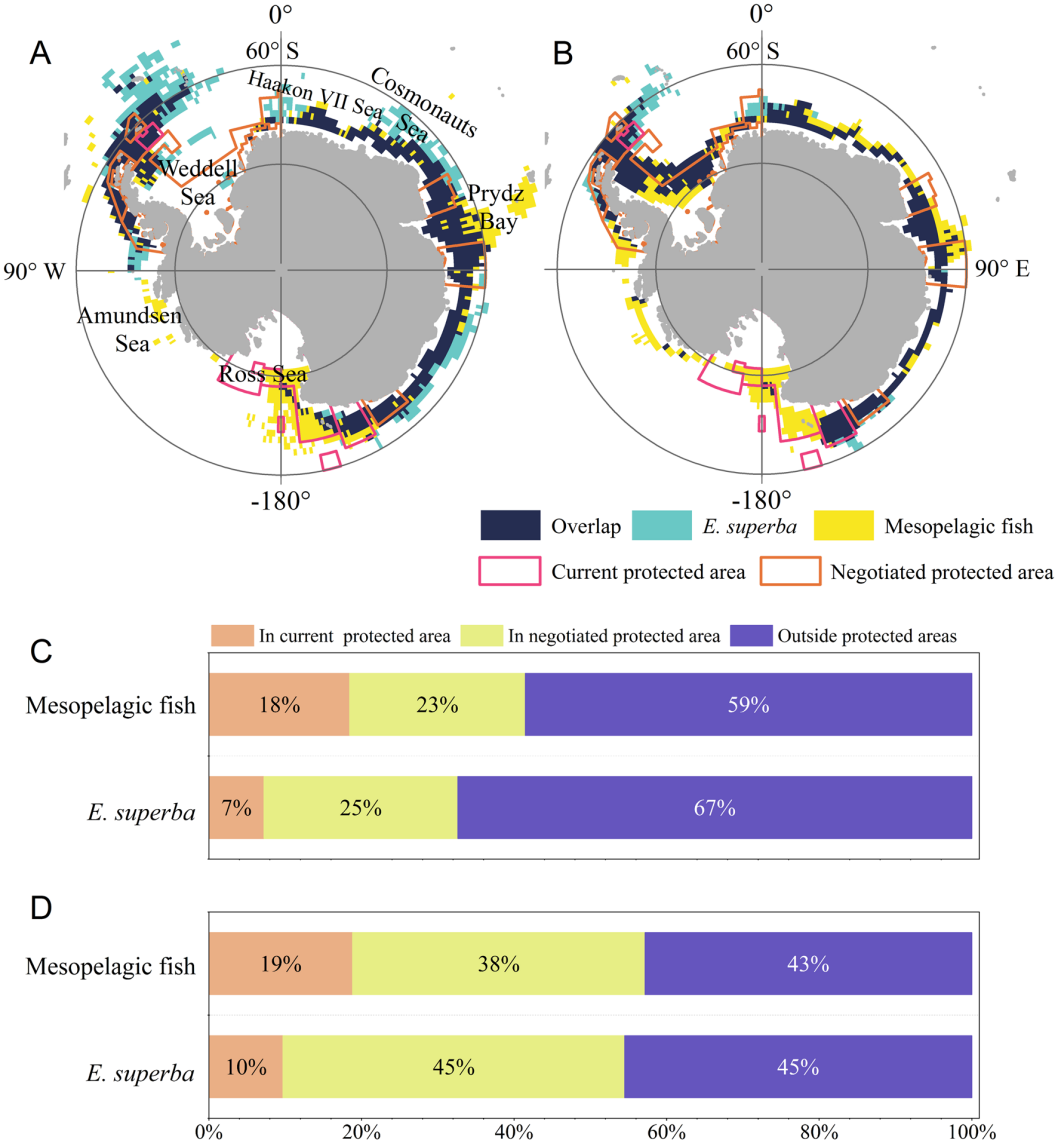
Important areas (IAs, top 5% HSI, habitat suitability index) of meso-fish and *E. superba* (krill) and their overlap regions in **A** present-day (1956–2005) and **B** RCP8.5 in long-term future (2050–2099). The magenta and orange lines represent current and negotiated protected area, respectively. The percentage area of IAs for meso-fish and *E. superba* (krill) in protected areas under **C** present-day (1956–2005) and **D** RCP8.5 in long-term future (2050–2099)

The present-day overlap of IAs for meso-fish and krill was mainly in the region northeast of the Antarctic Peninsula and the East Antarctic continental shelves, including Haakon VII Sea, Cosmonauts Sea, and Prydz Bay (Fig. 7A). In the long-term future, the IAs for meso-fish and krill were also partially covered by the protected areas (Fig. 7B). However, some IAs, such as Haakon VII Sea, Cosmonauts Sea, and Prydz Bay, are still not covered by these protected areas. At the present-day, the current protected areas cover 18% and 7% of the IAs for meso-fish and krill, respectively. The coverage of IAs by the negotiated protected areas for meso-fish and krill is 23% and 25%, respectively (Fig. 7C). In RCP8.5 (long-term future), the current protected areas would increase the coverage of IAs for meso-fish and krill to 19% and 10%, respectively (Fig. 7D). The coverage of IAs by the negotiated protected areas would increase to 38% and 45% for meso-fish and krill, respectively, especially with the proposed MPA in the Weddell Sea (Weddell Sea Marine Protected Area Phase1). In the present-day, IAs meso-fish and krill outside protected areas are 59% and 67%, respectively. In RCP8.5 (long-term future), this coverage would be reduced to 43% and 45%, respectively.

## Discussion

### Habitat distribution of mesopelagic fish in the SO

Species distribution models were constructed for nine meso-fish species in SO. These models use presence-only records to identify the baseline of these possible suitable (or unsuitable) habitats. For all meso-fish species, the environmental variable importance of SST and T_200 ranked the top three, indicating the pivotal roles of environmental impacts in the upper 200 m as the critical habitat. Meso-fishes have diurnal vertical migrations, staying in deep water during all or part of the day, and migrating to the upper water layers (surface to about 200 m) at night (Gjøsaeter and Kawaguchi 1980). Myctophids, such as *E. antarctica*, *G. braueri* and *G. nicholsi*, feed at night in the water layers above 200 m. In addition, *P. antarctica* occurs widely at night in waters above 200 m (Lancraft et al. 2004), whereas species such as *K. anderssoni* occupy the upper 200 m during the day (Duhamel et al. 2000, 2014; Koubbi et al. 2003; Lourenço et al. 2017).

Sea ice is the crucial environmental factor affecting the distribution of *N. coatsi*, *P. antarctica*, *B. antarcticus*, and *C. microdon*. It provides suitable feeding conditions, spawning and nursery habitats for the early life stages of species, such as *P. antarctica* (Agostini et al. 2015; Brierley and Thomas 2002). The proliferation of microorganisms and microalgae within and under sea ice drives primary production (Brierley and Thomas 2002). In this way, sea ice provides the essential habitat for zooplankton, especially copepods and amphipods that graze on sea ice algae. Many other organisms also depend on sea ice for reproduction and development throughout or at certain stages of their life cycles (Arndt and Swadling 2006). Meso-fish species consume predominantly zooplankton rather than phytoplankton, which may account for the limited influence of primary productivity on their distribution.

The nine meso-fish exhibit a present-day distribution consistent with known circumpolar patterns, including their association with water masses or fronts, and their preference or avoidance of the Antarctic shelf (Duhamel et al. 2014; Freer et al. 2019; Koubbi et al. 2003; La Mesa and Eastman 2012; Lourenço et al. 2017; Moteki et al. 2009; Ran et al. 2022; Woods et al. 2023). In the SO, the fronts may influence the distribution of pelagic fish by functioning as ecological barriers (Collins et al. 2012; Koubbi et al. 2003, 2011). The meso-fish distributed in the open ocean are mainly lanternfish, whose modeled habitats reflect the influence of oceanographic characteristics in structuring their spatially patchy, latitudinal distribution patterns (Duhamel et al. 2014; Freer et al. 2019; Koubbi et al. 2011). Lanternfish were all distributed south of the SAF, crossing the PF to reach the Antarctic continent. The SAF and PF are biogeographical boundaries, with pelagic organisms finding it more challenging to cross the vertical SAF, which often functions as a firm boundary. In contrast, the oblique PF, which acts more like a permeable boundary, seems easier to cross (Koubbi et al. 2003).

### Species association across meso-fish

Significant spatial relationships existed between the nine meso-fish species. The positive (or negative) correlations between species may indicate similar (or dissimilar) habitat requirements rather than direct or indirect interaction (e.g., symbiosis, competition) (Astarloa et al. 2019; Ovaskainen et al. 2010). Lanternfishes are known to coexist with other species, resulting in higher species associations compared to other meso-fish. Lanternfish and “high-Antarctic” species (*N. coatsi, P. antarctica, B. antarcticus*, and *C. microdon*) do not prefer the same habitat preferences. The distribution of lanternfish is closely related to the SAF and PF, the “high-Antarctic” species are distributed along the Antarctic continental shelf and slope.

Lanternfishes exhibit high spatial overlap and diverse diet, including copepods (*Metridia* spp., *Rhincalanus gigas*) and euphausiids (*Thysanoessa* spp., krill) (Saunders et al. 2022). The considerable dietary breadth of lanternfish is associated with differences in body size, changes in the depth of distribution, and migratory behavior among the species, such as observed among the common genera: *Electrona*, *Gymnoscopelus*, and *Protomyctophum* (Cherel et al. 2010; Shreeve et al. 2009). Although lanternfish overlap in their diets, differences in size, age, and distribution allow them to feed on diverse prey, and thus minimize competition. Inter-specific ecological niche segregation may reduce direct competition for prey, facilitate coexistence observed among *Gymnoscopelus* species (*G. braueri, G. nicholsi,* and *G. opisthopterus*) (Saunders et al. 2015b; Woods et al. 2020). *N. coatsi* and *B. antarcticus* were distributed in East Antarctica and the sea around the Antarctic Peninsula, showing high species association. The relatively low species association of *P. antarctica* and *C. microdon* may reflect different habitat requirements: *C. microdon* has a circum-Antarctic habitat, whereas *P. antarctica* is distributed in high Antarctic regions.

### Future habitat changes between eastern and western SO

Meso-fish in the SO are predicted to shift poleward under future scenarios, reducing the essential habitat. This conclusion is consistent with the other marine species’ responses to climate change (Fredston et al. 2021; Lenoir et al. 2011). Marine species may alternatively migrate deeper in response to climate change. However, this is unlikely for meso-fish as diel migration and larval stages depend on upper water (Duhamel et al. 2000, 2014; Freer et al. 2019; Gjøsaeter and Kawaguchi 1980; Koubbi et al. 2003; Lourenço et al. 2017). Although all nine meso-fish species would likely move poleward, the extent of habitat loss and poleward migration varied. In general, the further the poleward movement means more habitat loss. Lanternfish, such as *E. antarctica*, *G. opisthopterus,* and *G. braueri*, have narrow thermal niches and low physiological flexibility, making them more vulnerable to climate change and leading to future habitat loss (Freer et al. 2019).

Species habitat change differs between the western and eastern SO. These habitat changes may be explained by variations in environmental conditions (Supplementary Fig. S19). In the western SO near 60° S, increasing temperatures due to climate change would render the region unsuitable for meso-fish, especially lanternfish. In contrast, in areas of the Antarctic continental shelf and slope, SST and T_200 may not increase or may even decrease (Supplementary Fig. S19A–D). Sea ice is essential for *N. coatsi*, *P. antarctica*, *B. antarcticus*, and *C. microdon*. Across the SO, sea ice increased by 2.0 ± 0.4% per decade from 1979 to 2014. In addition, the Weddell Sea and the Ross Sea increased by 1.7 ± 0.9% and 4.3 ± 1.1% per decade, respectively, from 1979 to 2014, but the sea ice extent decreased in 2016 and slowly increased again in 2020 (Eayrs et al. 2021). Sea ice areas are predicted to increase in the Weddell Sea and the Ross Sea (Supplementary Fig. S19), which would allow *N. coatsi*, *P. antarctica*, *B. antarcticus*, and *C. microdon* to expand or maintain suitable habitats in these seas.

### Assessment for marine spatial planning

Protected areas overlap significantly with the habitat of meso-fish. Although current protected areas cover only 18% of meso-fish IAs, negotiated protected areas could increase coverage to 41%. This is consistent with findings from other taxa, e.g., proposed protected areas could cover 39% of ecologically significant areas for top predators (Hindell et al. 2020). These results support proposed MPAs in the SO and can inform ongoing and future assessments of established MPAs, such as the RSr MPA review in October 2022. Areas not included in protected areas around East Antarctica (e.g., Prydz Bay and the Cosmonauts Sea), the Antarctic Peninsula, and the Amundsen Sea were nonetheless ecologically significant for meso-fish. Shifts in IAs in the Weddell Sea over time underline the importance of the WSMPA Phase 1 proposal to future meso-fish distributions. Our results inform the design of new protected areas, highlighting the impact that conservation efforts combined with strictly defined rational use of fishing resources can promote scientific research and proactively tackle climate change.

To date, both established and proposed CCAMLR MPAs have not considered the diverse array of meso-fish (CCAMLR 2002, 2009, 2016, 2020a, b). The MPA design has prioritized the protection of predators, such as penguins, seals, whales, albatross, and Antarctic toothfish, as well as the protection of key species, namely, *P. antarctica*, and krill, and the promoting the protection of representative pelagic and benthic species assemblages, habitats, and ecosystems (CCAMLR 2002, 2009, 2016, 2020a, b). Meso-fish make an important contribution to the carbon cycle (Saba et al. 2021), they sequester carbon in the deep sea and mitigate climate change. Meso-fish are crucial to ecosystem stability by linking secondary producers to higher predators through prey–predator relationships and supporting the survival of thousands of other species (Murphy et al. 2007; Saunders et al. 2015a). Therefore, protecting meso-fish may contribute to the stability of the entire ecosystem, and protect the biodiversity of their predators and prey to some extent. The impacts of climate change on meso-fish could have severe consequences on both upper and lower trophic levels. Protecting meso-fish may mitigate the impacts of climate change on predators and prey. Neglecting meso-fish protection could negatively affect the upper and lower trophic species. Therefore, incorporating meso-fish into protected areas conservation objectives is essential. Including meso-fish in protected areas planning contributes to a better understanding of the whole ecosystem, and thus reduces uncertainties in protected areas planning analysis. Attention should be paid to their early life vulnerability, reproduction, ecosystem role, and suitable habitat connectivity to improve protected areas design and objectives.

Furthermore, shifts in meso-fish distributions due to climate change should be considered in protected areas design and assessment. These shifts could result in suitable habitats falling out of originally established/planned protected areas threatening previously protected species (Gilmour et al. 2022). When designing new protected areas, it is important to consider the potential economic and social implications as there may be trade-offs between conservation objectives and economic activities, e.g., fishing and tourism. We advocate for a comprehensive and integrated approach to marine conservation, which includes protected areas, climate change mitigation and adaptation strategies, sustainable fisheries management, and ecosystem-based management approaches. Tailored research and monitoring plans, coupled with regular reviews of protected areas, are needed to make protected areas designs responsive to such changes, adjusting boundaries as needed to maximize species protection and ecosystem benefits.

It should be noted that some environmental factors, such as the light environment, that potentially affect the fish's distribution, life history, and physiological processes may be ignored. Ambient light may limit the distribution of polar fish (Ljungstrom et al. 2021). The distribution of Antarctic meso-fish was stratified in different depths, and the same meso-fish species also had different distribution day and night (solar position) (Woods et al. 2023). In future studies, ambient light proxy and diel vertical migration may be explored to match the location of mesopelagic fish, which may be incorporated into the construction of SDM.

We utilized CMIP5 (Coupled Model Intercomparison Project Phase 5) data to explore meso-fish distribution. However, future research should explore the impact of CMIP6 data more extensively. The input of future data environmental variables determines the reliability of species distribution predictions. Although many studies have discussed the differences between CMIP5 and CMIP6 models (Carvalho et al. 2022), only a few have investigated their distinct effects on predicting species distribution.

The ESMs variability may lead to the uncertainty of the projected distribution of meso-fish (Freer et al. 2019). The simulations from a single model may lead to misleading or uninformative predictions, so the principal suggestions from some reviews include that ecologists should construct multi-model simulations and multi-RCP to capture the uncertainty (Freer et al. 2018). The results of this study are the ensemble average of the predictions incorporated multi-SDM algorithms, multi-ESMs simulations, and multi-RCP scenarios. It is impossible to eliminate uncertainty but this study reduces uncertainty by the ensemble approach.

The biotic variables influenced the distribution of meso-fish; these species interactions, potential evolutionary processes, and other anthropogenic impacts were ignored in this research. The species around the Antarctic may adapt to the environmental variations. Some species may shift their suitable thermal range for adapting to climate change, even increasing distribution and abundance (Guerra et al. 2021). This may lead to uncertainty in the future distribution of meso-fish.

## Materials and methods

### Species occurrence records

Biological data used in this study are species occurrence records (presence-only) of nine meso-fish species: *Electrona antarctica* (Myctophidae; Antarctic lanternfishes), *Krefftichthys anderssoni* (Myctophidae; Rhombic lanternfish), *Gymnoscopelus braueri* (Myctophidae; Brauer’s lanternfish), *Notolepis coatsi* (Paralepididae; Antarctic jonafish), *Gymnoscopelus nicholsi* (Myctophidae; Nichol's lanternfish), *Gymnoscopelus opisthopterus* (Myctophidae), *Pleuragramma antarctica* (Nototheniidae: Antarctic silverfish), *Bathylagus antarcticus* (Bathylagidae: Antarctic deepsea smelt), *Cyclothone microdon* (Gonostomatidae; Veiled anglemouth). These species belong to the four most abundant fish families in meso-fish (Duhamel et al. 2014; Woods et al. 2022). These nine species are widely distributed in the SO and thus well-represent the SO meso-fish community (Duhamel et al. 2014; Koubbi et al. 2011; Moteki et al. 2009; Saunders et al. 2015b).

The occurrence records of the nine meso-fish species collected from the Global Biodiversity Information Facility (GBIF, http://www.gbif.org), the Ocean Biodiversity Information System (OBIS, http://www.iobis.org), and published literature (Freer et al. 2019). In addition, occurrence records of Antarctic krill (*Euphausia superba*) were obtained from KRILLBASE (Atkinson et al. 2017), OBIS, and GBIF.

Only occurrence records from 1955 onwards and within the Southern Ocean (South of 35° S) were used for further analysis to align the biological data with the environmental variables. Pre-processing and data exploration was conducted for each species by (i) removing unreliable records, such as preserved specimens and terrestrial records; (ii) discarding duplicated records; and (iii) rarefying records spatially by taking a mean value for cells with multiple records to ensure only one record occurred in each 1° grid cell. The occurrence records correspond with the spatial resolution of the environmental variables to prevent sampling bias (Syfert et al. 2013). Photographs of the nine meso-fish and the distribution of occurrence records of the nine meso-fish are shown in Supplementary Fig. S1. Krill occurrence is shown in Supplementary Fig. S2.

### Environmental variables

Environmental variables of the surface and 200 m depth level have been well-documented in the distribution of most meso-fishes (Duhamel et al. 2014; Koubbi et al. 2011; Loots et al. 2007; Ran et al. 2022). Based on analyses of the physiological characteristics, data availability, and summaries, seven environmental variables were included to construct SDMs. These were (i) sea ice area fraction, (ii) SST, (iii) SSS, (iv) T_200, (v) S_200, (vi) depth, and (vii) primary productivity. Depth data were retrieved from the global relief model ETOPO1 (https://www.ngdc.noaa.gov/mgg/global/). The other six environmental variables were derived from the Coupled Model Intercomparison Project Phase 5 (CMIP5) ESM. A summary of the seven environmental variables is detailed in Table 1. All environmental variables were resampled to a spatial resolution of 1° using the bilinear resample method. Because a single subset of ESM may not provide sufficient data, environmental variables from different ESMs could lead to different distribution predictions (Freer et al. 2018). Environmental variables from different ESMs were used to model the species distribution. Climate simulations were based on RCP scenarios of the Intergovernmental Panel on Climate Change (IPCC). RCP scenarios were used usually to project species distribution (Freer et al. 2018, 2019, 2023; Hindell et al. 2020; Liu et al. 2020; Ran et al. 2022). Climate simulations from all ESMs were considered under the medium emission scenario (RCP4.5) and the pessimistic scenario (RCP8.5). Only ESMs, including the six environmental variables under RCP4.5 and RCP8.5, were used for further analysis, i.e., CESM1–BGC, GFDL–ESM2G, GFDL–ESM2M, and HadGEM2-ES (Supplementary Tables S1 and S2).

**Table 1:** Environmental variables used for model construction

Variable (unit)	Name	Spatial resolution/°	Source	Data acquisition
SST (°C)	Sea surface temperature	1	BGC, E2G, E2M, ES	https://psl.noaa.gov/ipcc/ocn/
T_200 (°C)	Temperature at 200 m	1	BGC, E2G, E2M, ES	https://psl.noaa.gov/ipcc/ocn/
SSS	Sea surface salinity	1	BGC, E2G, E2M, ES	https://psl.noaa.gov/ipcc/ocn/
S_200	Salinity at 200 m	1	BGC, E2G, E2M, ES	https://psl.noaa.gov/ipcc/ocn/
Sea ice (%)	Sea ice fractional coverage	1	BGC, E2G, E2M, ES	https://psl.noaa.gov/ipcc/ocn/
Primary productivity	Primary organic carbon production	1	BGC, E2G, E2M, ES	https://psl.noaa.gov/ipcc/ocn/
Depth (m)	Bathymetry	1/60	Etopo1 Global Relief Model	https://www.ngdc.noaa.gov/mgg/global/

*BGC* CESM1–BGC, *E2G* GFDL–ESM2G, *E2M* GFDL–ESM2M, *ES* HadGEM2-ES

Based on the time range of environmental variables from four ESMs, for the present-day (1956–2005), the six environmental variables were obtained from the four ESMs. For the future climate simulations (RCP4.5 and RCP8.5), two time periods, i.e., short-term future (2006–2055) and long-term future (2050–2099), were derived from each of the four ESMs. Here, we discuss mainly the RCP8.5 results, because current emissions are similar or slightly higher than the RCP8.5 scenario (Peters et al. 2013).

### Species distribution modeling

RF, BRT, and MAXENT were used to model species distribution by fitting occurrence records and environmental variables. These three SDMs are commonly used to predict species distribution in the SO (Duhamel et al. 2014; Freer et al. 2019; Hindell et al. 2020; Pinkerton et al. 2010; Ran et al. 2022; Woods et al. 2023). No environmental variables were excluded from the models due to collinearity for the following reasons: (1) machine learning (RF, BRT, and Maxent) can effectively deal with collinearity and account for the complex interactions among environmental variables (Charlène et al. 2020; Ellis et al. 2012; Hapfelmeier et al. 2014; Phillips et al. 2006); and (2) Including more biologically relevant predictors often results in better predictive performance (Duhamel et al. 2014; Freer et al. 2019; Hindell et al. 2020; Pinkerton et al. 2010; Ran et al. 2022; Xavier et al. 2016).

All SDMs were repeated 10 times using the cross-validation method, with 70% of the data used for training and 30% for testing. The predictive performance of the models was evaluated using two performance metrics, the TSS and the AUC. The AUC ranges from 0 to 1, and the TSS ranges from – 1 to 1. A value of 1 for both AUC and TSS represents perfect predictive performance (Allouche et al. 2006). We constructed an RF, BRT, and MAXENT ensemble model using the TSS values as a weighting factor. In ensemble models, the R package “SDM” uses presence-only data and environmental variables to compute the habitat suitability index (HSI) (Naimi and Araújo 2016). HSI values closer to or equal to 1 indicate habitats with high potential (or high probability) (Naimi and Araújo 2016). The threshold of the top 5% of the habitat distribution was used to transform continuous habitat suitability into the binary distribution of suitable/unsuitable habitats (e.g., a value of 0.95 or higher represents the top 5% of the habitat distribution) (Sillero et al. 2021). The variable importance was calculated using the function “getVarImp” in the “SDM” R package (Naimi and Araújo 2016).

For each species, the environmental variables of the present-day and the future climate simulations (short-term and long-term future) for the four ESMs were used to predict the present and future distribution. Thus, four sets of present-day predictors and 16 sets of future climate simulations (four ESMs of RCP4.5 for each of the two periods and four ESMs of RCP8.5 for each) were used as input to the SDM. Each species’ current and future distribution maps were ensemble-averaged from each of the four predictions of ESMs to obtain a robust output.

### Species association analysis

Species distribution patterns may be attributed to environmental responses and drivers of latent variables, the latter of which can be understood as unobserved predictors inducing correlations between species (Astarloa et al. 2019). Residual correlation from latent variables may represent species correlations using the “jSDM” R package (Clément and Vieilledent 2022). Therefore, we calculated the residual correlation using binary habitat and environmental variables (derived from the four ESMs) to explore species correlation among the nine meso-fish. To determine if the residual correlations were “significant”, 99% highest posterior density intervals were used. The residual correlation was ensemble-averaged from the four ESMs to obtain averaged species associations.

### Analysis of habitat distribution under climate change

The habitat maps of each species were re-projected to the Lambert Azimuthal equal-area (South Pole) to avoid potential bias due to unequal cell sizes (Budic et al. 2016). To describe changes in habitat distribution, habitat loss (i.e., habitat reduction), stability (i.e., no change in habitat), and gain (i.e., habitat range expansion) of each species was calculated by comparing the binary habitat of the future climate simulations and present-day (Brown et al. 2017). To better understand habitat changes, centroid changes in the suitable habitat distribution of each species were also calculated between present-day and future climate simulations. Robust centroid changes and habitat area results for each species were ensemble-averaged from the four ESMs outputs for each RCP and time period.

### Assessing overlap of the important areas with protected areas

To identify the IAs for meso-fish, the habitat overlap of the nine meso-fish was ensemble-averaged to obtain the mean HSI (Naimi and Araújo 2016) of each meso-fish. Then, the top 5% habitat threshold was used to identify IAs (Hindell et al. 2020). The IAs of Antarctic krill were identified also using this threshold. In addition to the two MPAs already established (SOISS and RSr MPAs), three MPAs are currently proposed: Domain 1 Marine Protected Area, East Antarctic Representative System of Marine Protected Areas, and the Weddell Sea Marine Protected Area Phase1. For more details see CCAMLR MPA Information Repository (https://cmir.ccamlr.org/). The overlap of these regions was calculated to evaluate the extent to which the IAs for meso-fish and Antarctic krill are covered by current and negotiated protected areas.

### Supplementary Information

Below is the link to the electronic supplementary material.

Supplementary file1 (PDF 3062 KB)

## Data Availability

The authors will make the raw data and code supporting this article available without undue reservation.
