# Correction: Incorporating mesopelagic fish into the evaluation of marine protected areas under climate change scenarios

**DOI:** 10.1007/s42995-023-00193-y

**Published:** 2023-10-13

**Authors:** Shuhao Liu, Yang Liu, Katharina Teschke, Mark A. Hindell, Rachel Downey, Briannyn Woods, Bin Kang, Shuyang Ma, Chi Zhang, Jianchao Li, Zhenjiang Ye, Peng Sun, Jianfeng He, Yongjun Tian

**Affiliations:** 1 Research Centre for Deep Sea and Polar Fisheries, and Key Laboratory of Mariculture, Ministry of Education, Ocean University of China, Qingdao, 266003 China; 2 Frontiers Science Center for Deep Ocean Multispheres and Earth System, Ocean University of China, Qingdao, 266100 China; 3 Alfred Wegener Institute, Helmholtz Centre for Polar and Marine Research, Am Handelshafen 12, 27570 Bremerhaven, Germany; 4 Helmholtz Institute for Functional Marine Biodiversity at the University Oldenburg, Ammerländer Heerstraße 231, 23129 Oldenburg, Germany; 5 Institute for Marine and Antarctic Studies, University of Tasmania, Hobart, 7004 Australia; 6 Fenner School of Environment and Society, Australian National University, Canberra, ACT 2602 Australia; 7 College of Fisheries, Ocean University of China, Qingdao, 266003 China; 8 Polar Research Institute of China, Shanghai, 200136 China

**Correction: Marine Life Science & Technology** 10.1007/s42995-023-00188-9

The author wants to highlight the important role of mesopelagic fish in the conservation of marine living resources. Therefore, in order to further clarify and focus research point on the conservation of marine living resources, the authors replaced the MPA with the protected areas, and slightly modified several other vague places. In these corrections, most of them are the replacement of the terms.

The original article has been corrected.

The text “marine protected areas” is replaced with “conservation areas for marine living resources” in the title, Line 2–3.

The text "the Marine protected areas" is replaced with "mesopelagic fish" in Line 24.

The text "the MPA with protected areas" is replaced in Line 10, Line 23, Line 359, Line 370, Line 508, Line 519, Line 546, Line 547, Line 549, Line 552, Line 554, Line 556, Line 558, Line 562, Line 566, Line 769,

Several vague places using protected areas are slightly modified in Line 15, Line 20, Line 21, Line 22, Line 155–156, Line 331–332, Line 356–357, Line 359, Line 361, Line 363, Line 365–366, Line 504, Line 505, Line 506, Line 513, Line 783–784.

For replacement of the MPA using protected areas, the Fig. 7 and legend are modified without the change of the numbers.
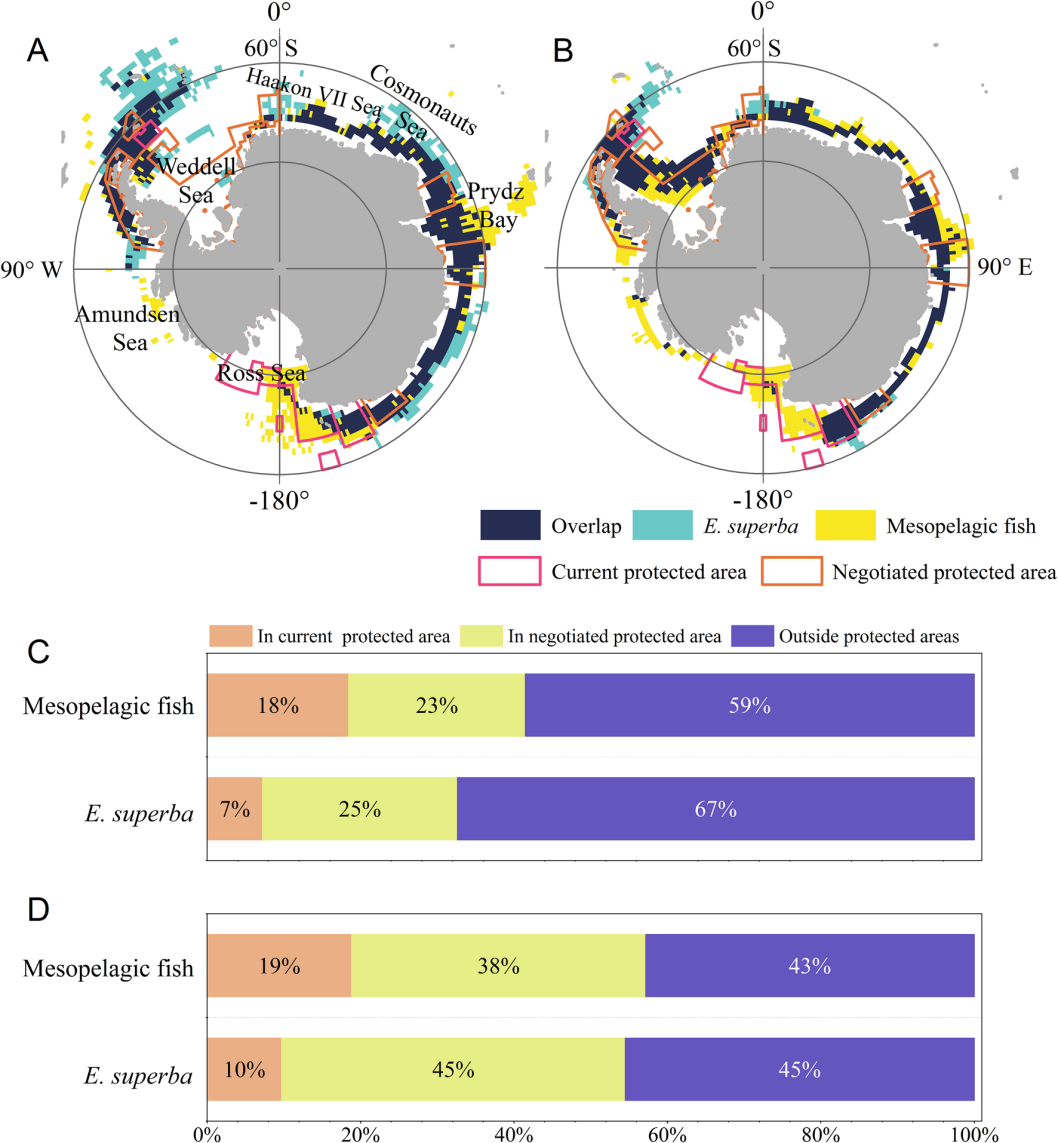


The text “marine protected areas” is replaced with “conservation areas for marine living resources” in the title in Supplementary information.

