# Supplementary material for: Incorporating mesopelagic fish into the evaluation of conservation areas for marine living resources under climate change scenarios
Source: Mar Life Sci Technol. 2023 Aug 15;6(1):68–83. doi: 10.1007/s42995-023-00188-9 (PMC10902249; doi:10.1007/s42995-023-00188-9)
Supplement: Supplementary file 1 — Supplementary file1 (PDF 3062 KB) [file 42995_2023_188_MOESM1_ESM.pdf]

Incorporating mesopelagic fish into the evaluation of conservation areas for marine living resources under climate change scenarios

Shuhao Liu[1], Yang Liu[1,2], Katharina Teschke[3,4], Mark A. Hindell[5], Rachel Downey[6], Briannyn Woods[5], Bin Kang[7], Shuyang Ma[1], Chi Zhang[1], Jianchao Li[1], Zhenjiang Ye[1], Peng Sun[1], Jianfeng He[8], Yongjun Tian[1,2]

[1]Research Centre for Deep Sea and Polar Fisheries, and Key Laboratory of Mariculture, Ministry of Education, Ocean University of China, Qingdao 266003, China

[2]Frontiers Science Center for Deep Ocean Multispheres and Earth System, Ocean University of China, Qingdao 266100, China

[3]Alfred Wegener Institute, Helmholtz Centre for Polar and Marine Research, Am Handelshafen 12, Bremerhaven 27570, Germany

[4]Helmholtz Institute for Functional Marine Biodiversity at the University Oldenburg, Ammerländer Heerstraße 231, Oldenburg 23129, Germany

[5]Institute for Marine and Antarctic Studies, University of Tasmania, Hobart 7004, Australia

[6]Australian National University, Fenner School of Environment & Society, Canberra, ACT 2602, Australia

[7]College of Fisheries, Ocean University of China, Qingdao 266003, China

[8]Polar Research Institute of China, Shanghai 200136, China

Correspondence

Yang Liu, Research Centre for Deep Sea and Polar Fisheries, and Key Laboratory of Mariculture, Ministry of Education, Frontiers Science Center for Deep Ocean Multispheres and Earth System, Ocean University of China, Qingdao, China.

E-mails: yangliu315@ouc.edu.cn

Marine Life Science and Technology

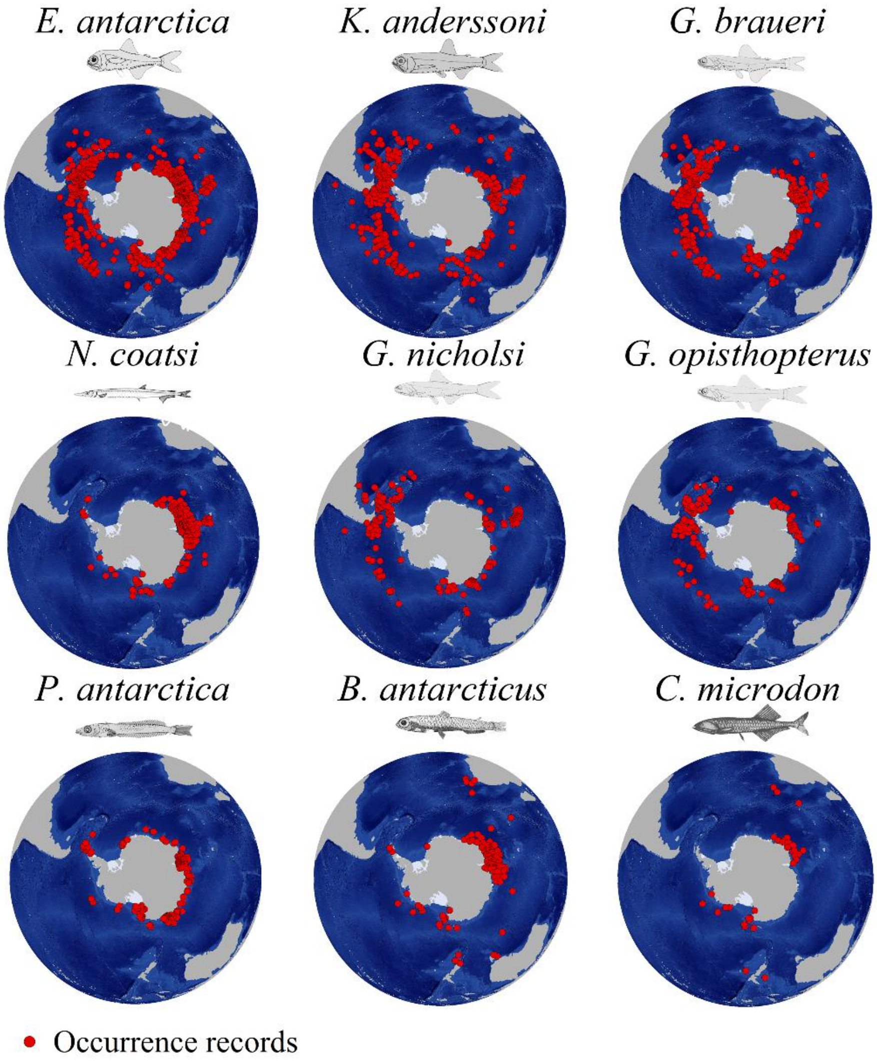

Figure S1. The pictures and distribution maps of the occurrence records of the nine mesopelagic fish are considered in this study. The pictures of the nine mesopelagic fish were obtained from https://www.fishbase.se/search.php.

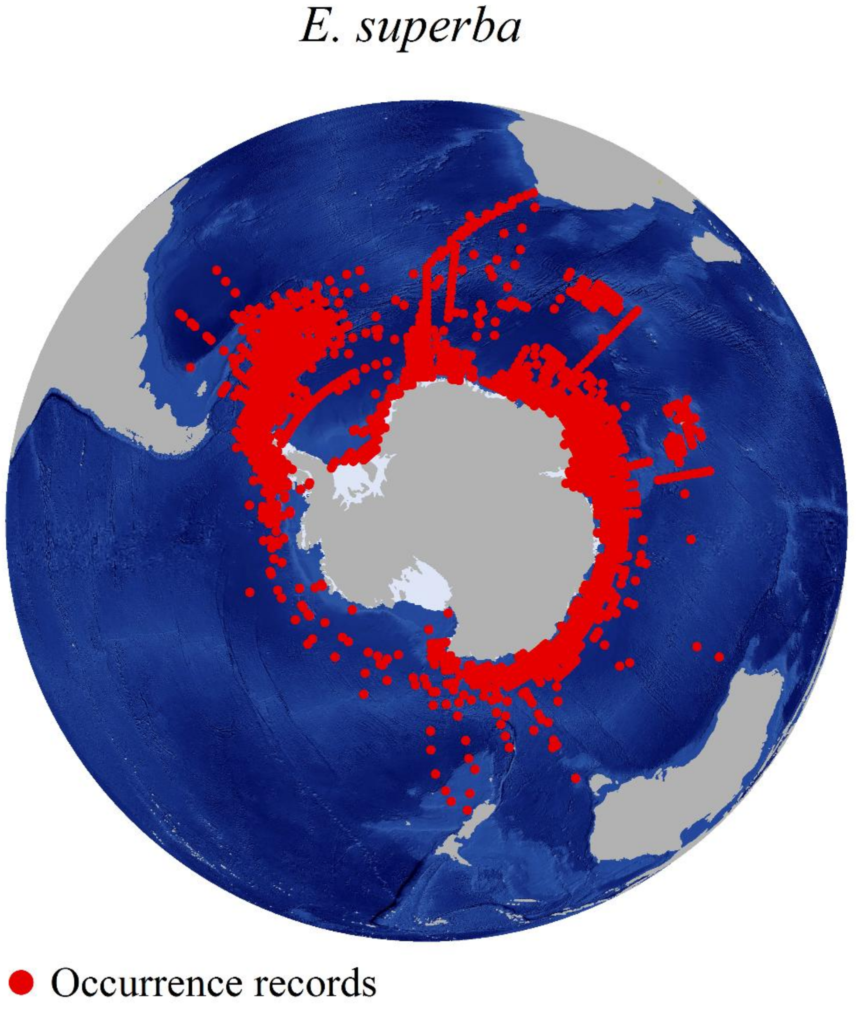

Figure S2. The distribution of occurrence records Antarctic krill (*Euphausia superba*).

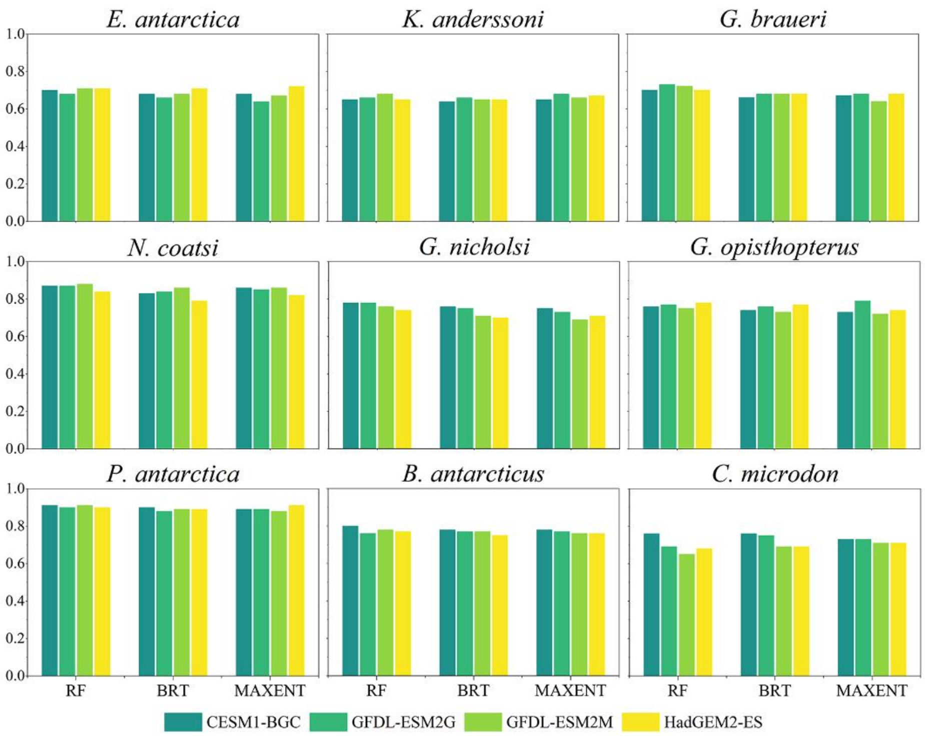

Figure S3. Averaged performance metrics (TSS: true skill statistics) with 10 runs for random forest (RF), boosted regression tree (BRT), and maximum entropy (MAXENT). The environmental variables used for modeling were from four Earth System Models (CESM1-BGC, GFDL-ESM2G, GFDL-ESM2M, and HadGEM2-ES).

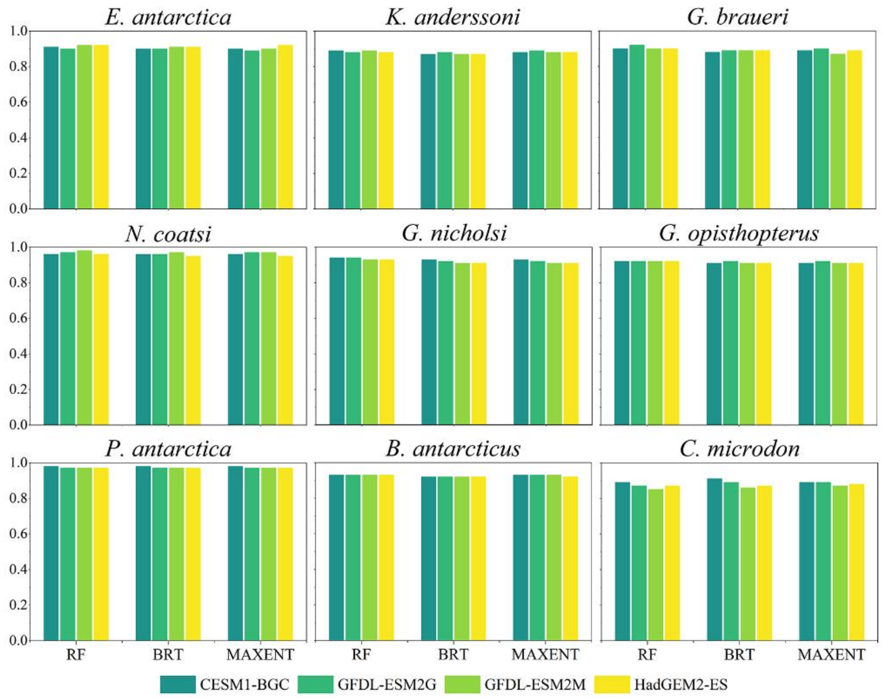

Figure S4. Averaged performance metrics (AUC: area under the receiver operating characteristic curve) with 10 runs for random forest (RF), boosted regression tree (BRT), and maximum entropy (MAXENT). The environmental variables used for modeling were from four Earth System Models (CESM1-BGC, GFDL-ESM2G, GFDL-ESM2M, and HadGEM2-ES).

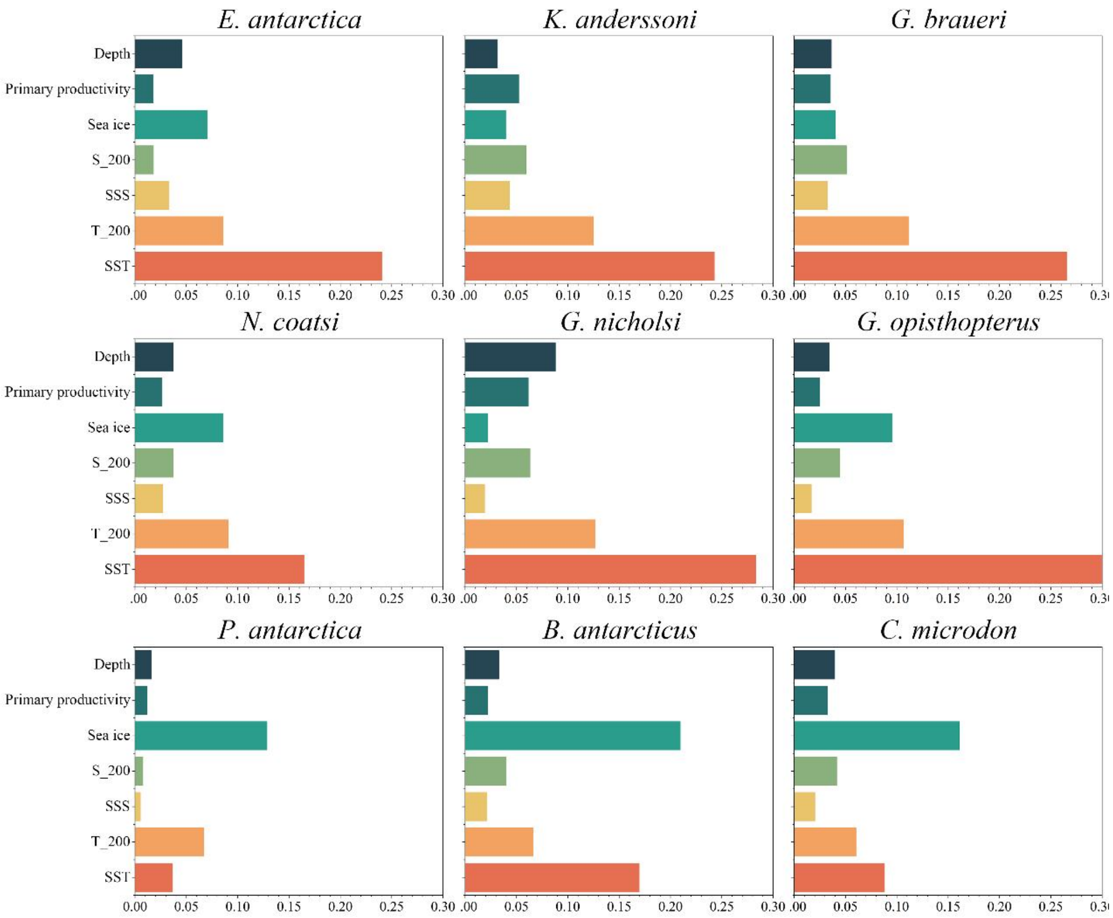

Figure S5. Averaged variable importance of the seven environmental variables for all nine mesopelagic fish from four Earth System Models (CESM1-BGC, GFDL-ESM2G, GFDL-ESM2M, and HadGEM2-ES). (SST: Sea surface temperature; T_200: the temperature at 200m; SSS: sea surface salinity; S_200; salinity at 200m, Sea ice is the fractional coverage of a grid cell that is covered with sea ice).

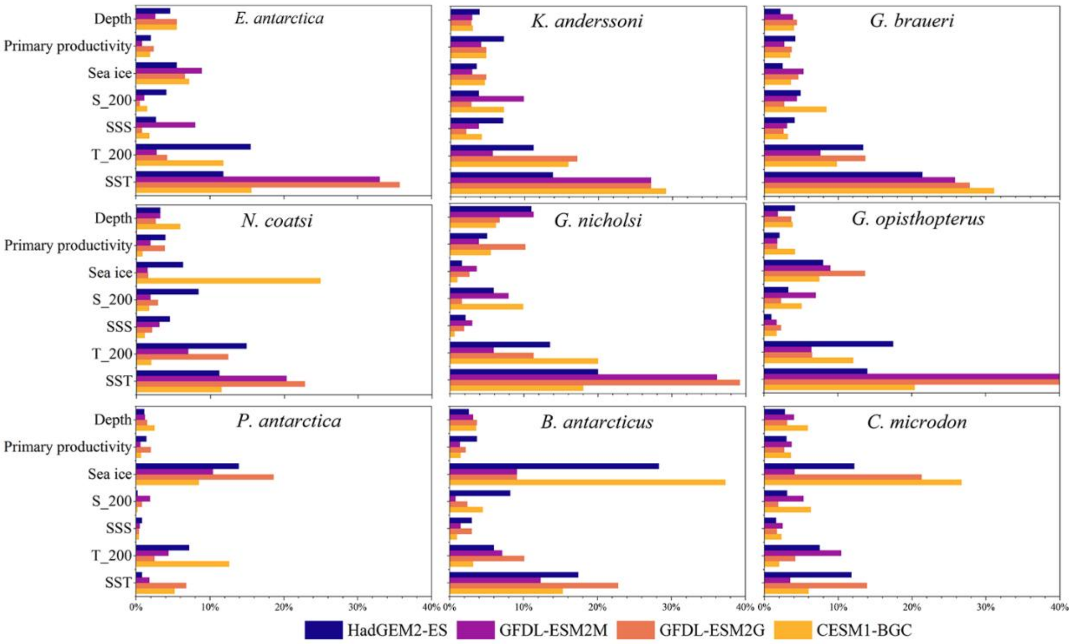

Figure S6. Variable importance of nine mesopelagic fish based on seven environmental variables based on four Earth System Models (CESM1-BGC, GFDL-ESM2G, GFDL-ESM2M, HadGEM2-ES). (SST: Sea surface temperature; T_200: the temperature at 200m; SSS: sea surface salinity; S_200; salinity at 200m, Sea ice is the fractional coverage of a grid cell that is covered with sea ice).

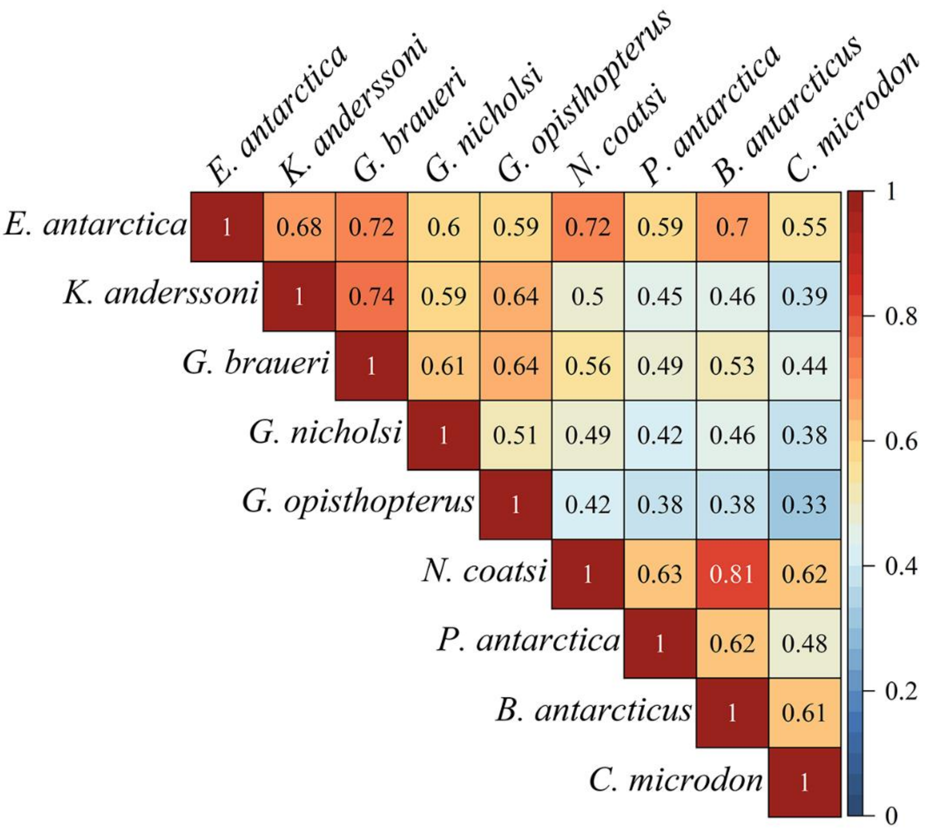

Figure S7. The species associations measured residual correlation for nine mesopelagic fish based on CESM1-BGC of Earth System Models. The associations have significant correlations (whose 99% highest posterior density interval).

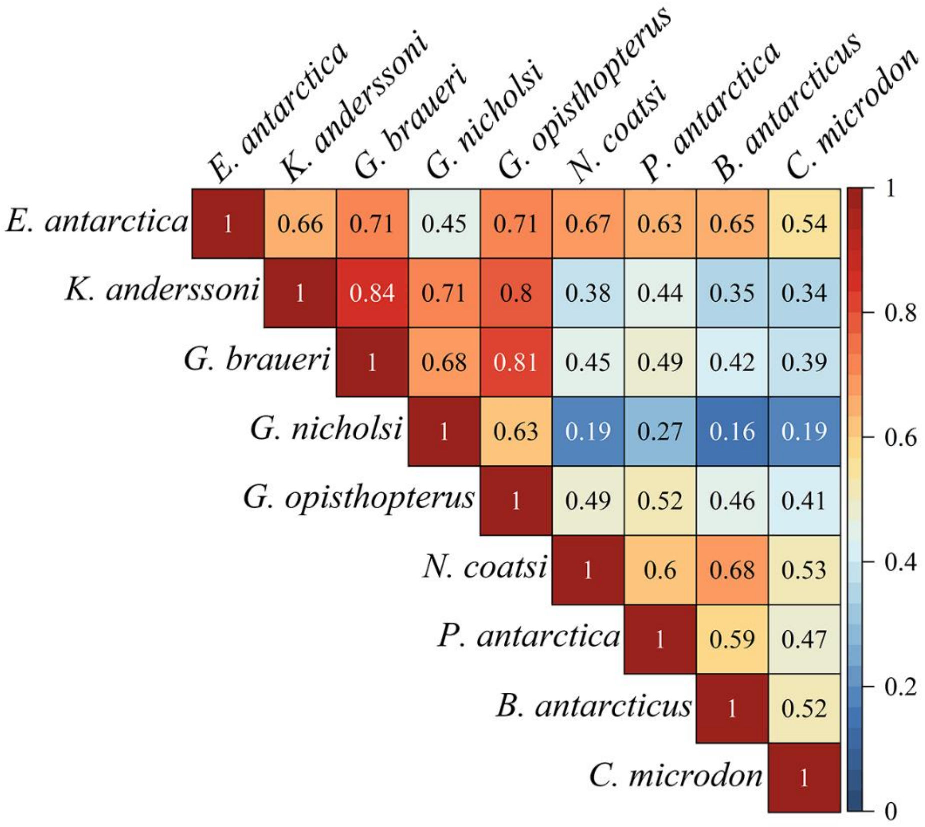

Figure S8. The species associations measured residual correlation for nine mesopelagic fish based on GFDL-ESM2G of Earth System Models. The associations have significant correlations (whose 99% highest posterior density interval).

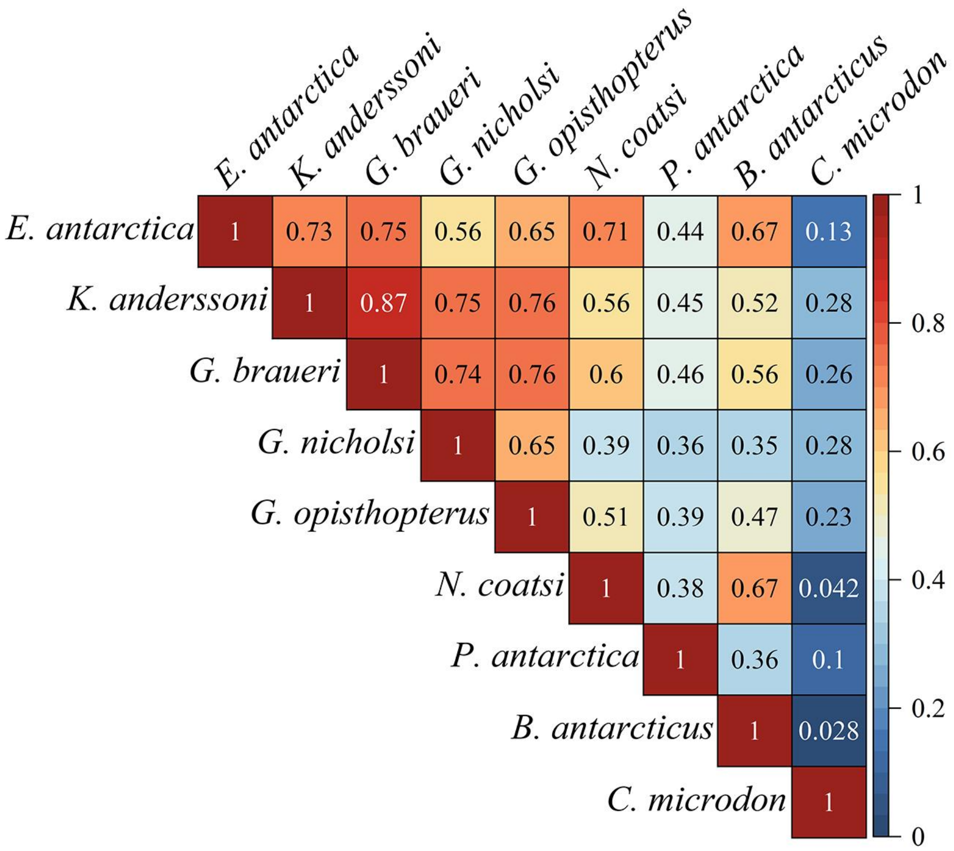

Figure S9. The species associations measured residual correlation for nine mesopelagic fish based on GFDL-ESM2M of Earth System Models. The associations have significant correlations (whose 99% highest posterior density interval).

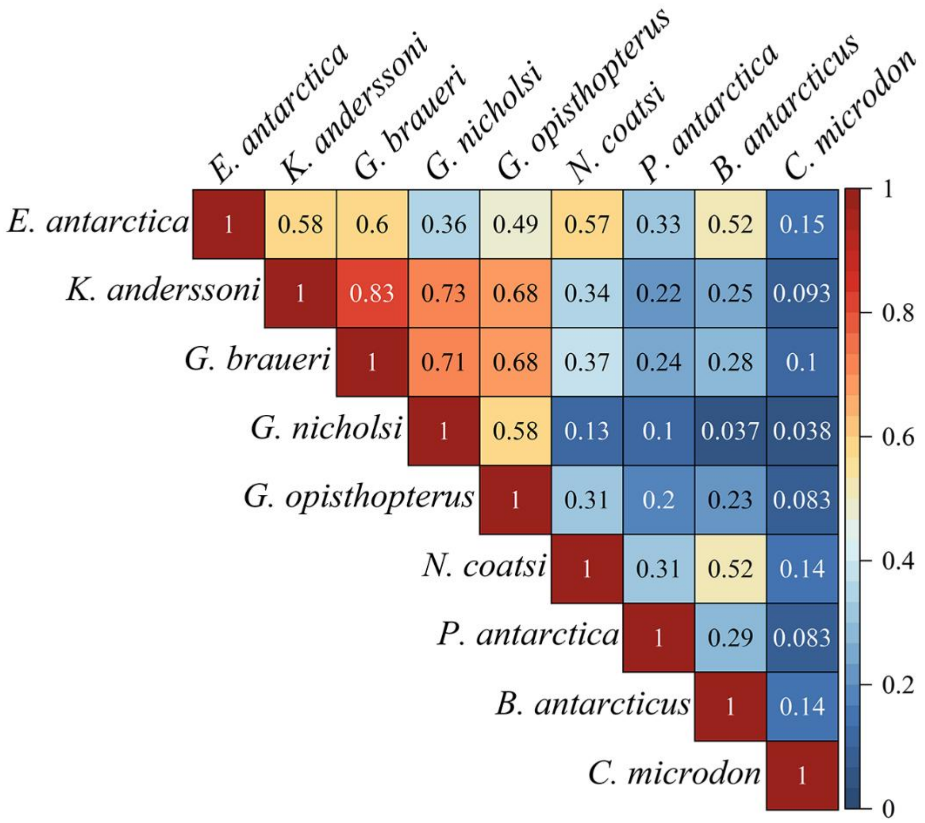

Figure S10. The species associations measured of residual correlation for nine mesopelagic fish based on HadGEM2-ES of Earth System Models. The associations have significant correlations (whose 99% highest posterior density interval).

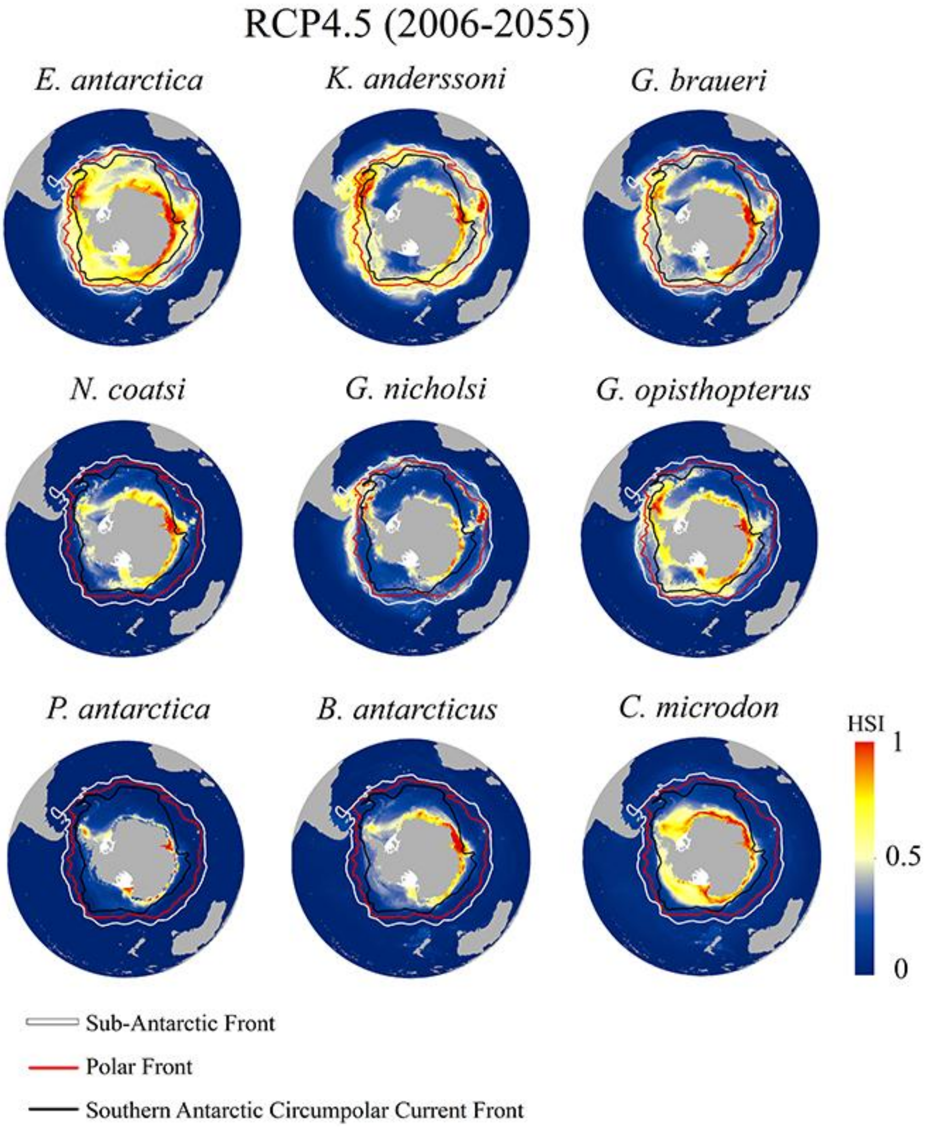

Figure S11. The future distribution of habitat suitability index (HSI) for nine mesopelagic fish by 2006-2055 under RCP4.5. The main oceanographic fronts are shown: sub-Antarctic Front (SAF; white line), Polar Front (PF; red line), and Southern Antarctic Circumpolar Current Front (SACCF; black line). The oceanographic front data from Australian Antarctic Data Centre. Representative Concentration Pathway scenarios: RCP.

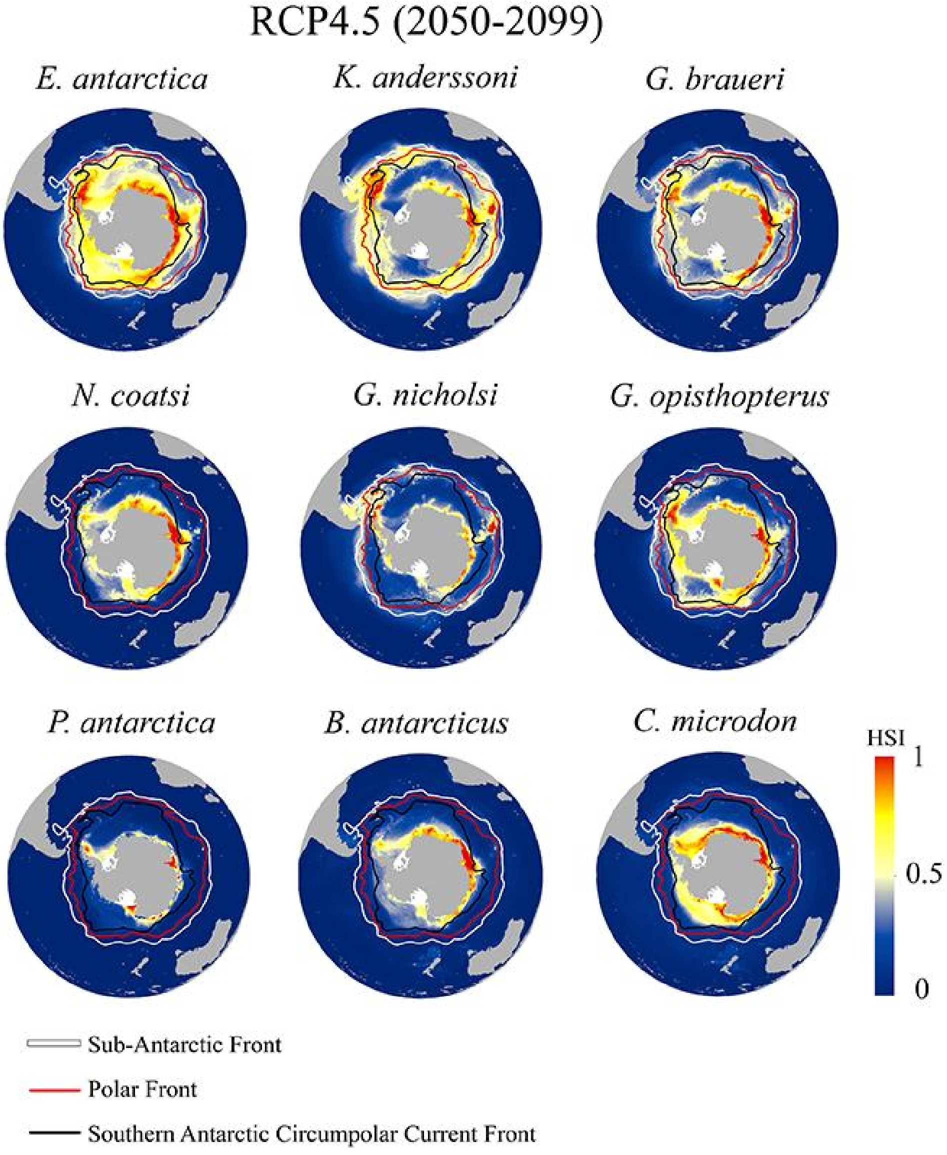

Figure S12. The future distribution of habitat suitability index (HSI) for nine mesopelagic fish by 2050-2099 under RCP4.5. The main oceanographic fronts are shown: sub-Antarctic Front (SAF; white line), Polar Front (PF; red line), and Southern Antarctic Circumpolar Current Front (SACCF; black line). The oceanographic front data from Australian Antarctic Data Centre. Representative Concentration Pathway scenarios: RCP.

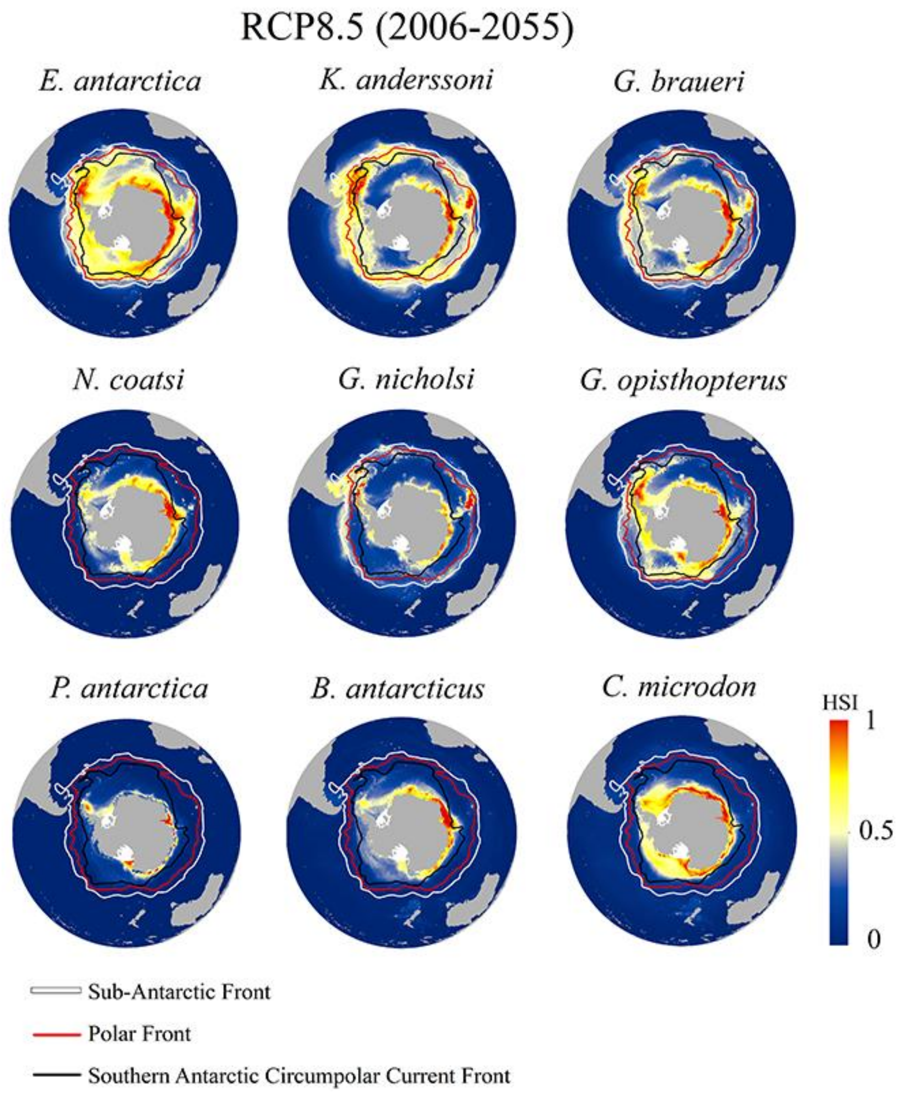

Figure S13. The future distribution of habitat suitability index (HSI) for nine mesopelagic fish by 2006-2055 under RCP8.5. The main oceanographic fronts are shown: sub-Antarctic Front (SAF; white line), Polar Front (PF; red line), and Southern Antarctic Circumpolar Current Front (SACCF; black line). The oceanographic front data from Australian Antarctic Data Centre. Representative Concentration Pathway scenarios: RCP.

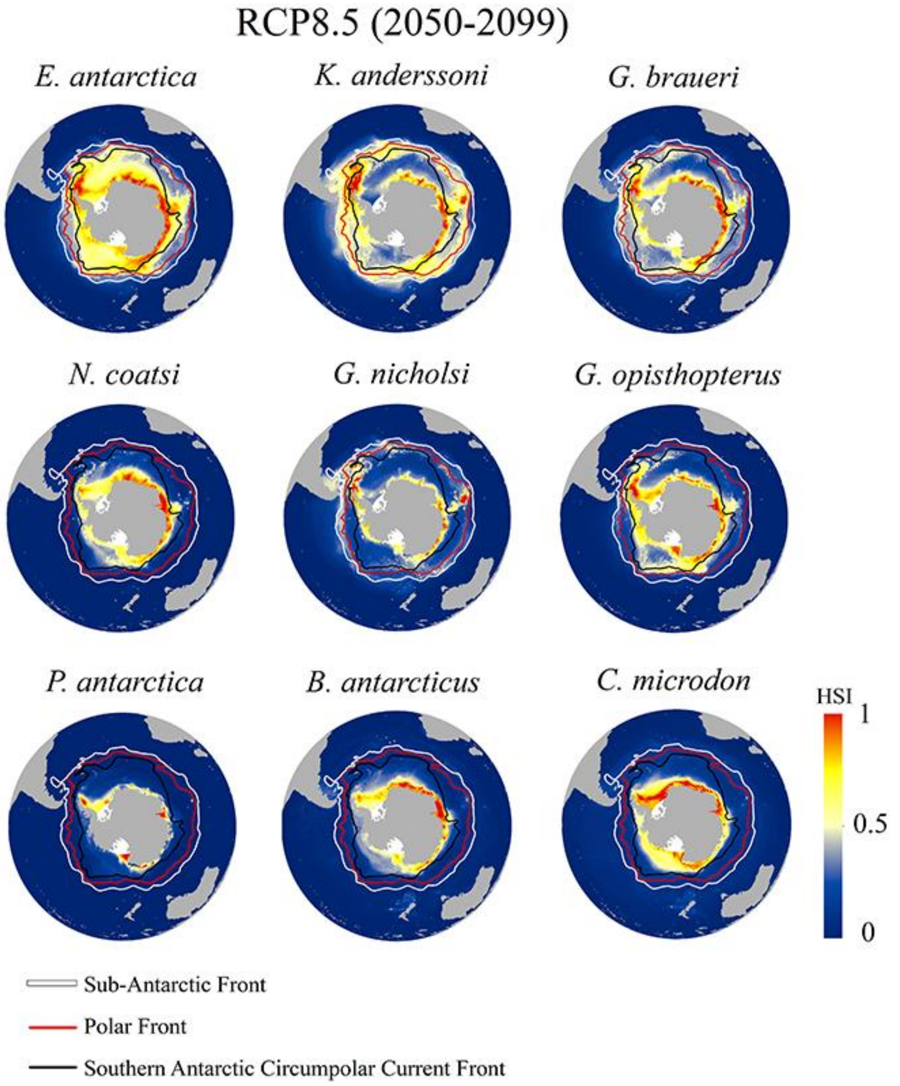

Figure S14. The future distribution of habitat suitability index (HSI) for nine mesopelagic fish by 2050-2099 under RCP8.5. The main oceanographic fronts are shown: sub-Antarctic Front (SAF; red line), Polar Front (PF; green line), and Southern Antarctic Circumpolar Current Front (SACCF; black line). The oceanographic front data from Australian Antarctic Data Centre. Representative Concentration Pathway scenarios: RCP.

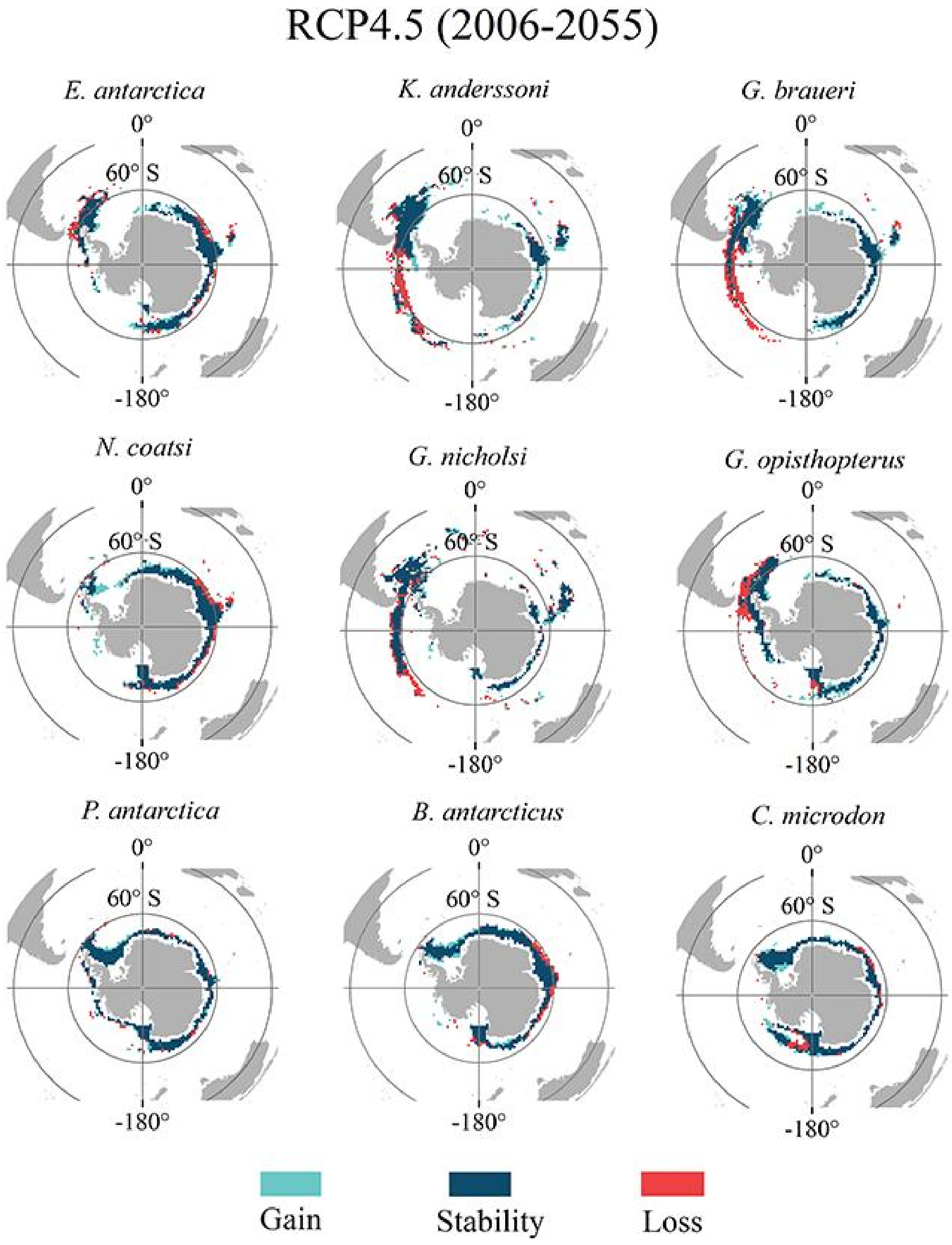

Figure S15. The habitat change in the predicted distribution for nine mesopelagic fish by 2006-2055 under medium emission scenario RCP4.5 relative to 1956-2005. The light blue, dark blue, and red areas represent habitat gain (habitat range expansion), habitat stability (no change in habitat), and habitat loss (habitat range contraction), respectively. Representative Concentration Pathway scenarios: RCP.

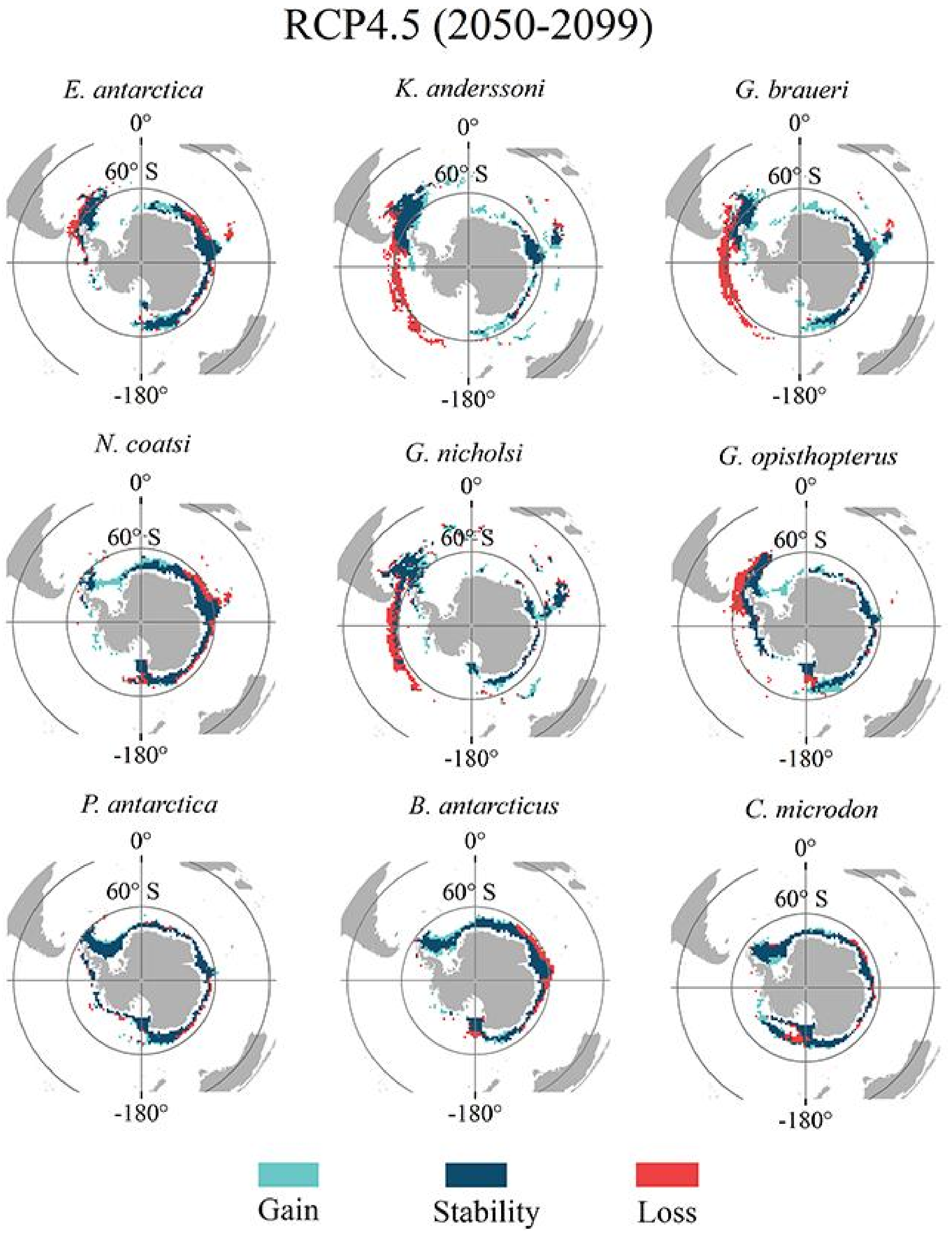

Figure S16. The habitat change in the predicted distribution for nine mesopelagic fish by 2050-2099 under medium emission scenario RCP4.5 relative to 1956-2005. The light blue, dark blue, and red areas represent habitat gain (habitat range expansion), habitat stability (no change in habitat), and habitat loss (habitat range contraction), respectively. Representative Concentration Pathway scenarios: RCP.

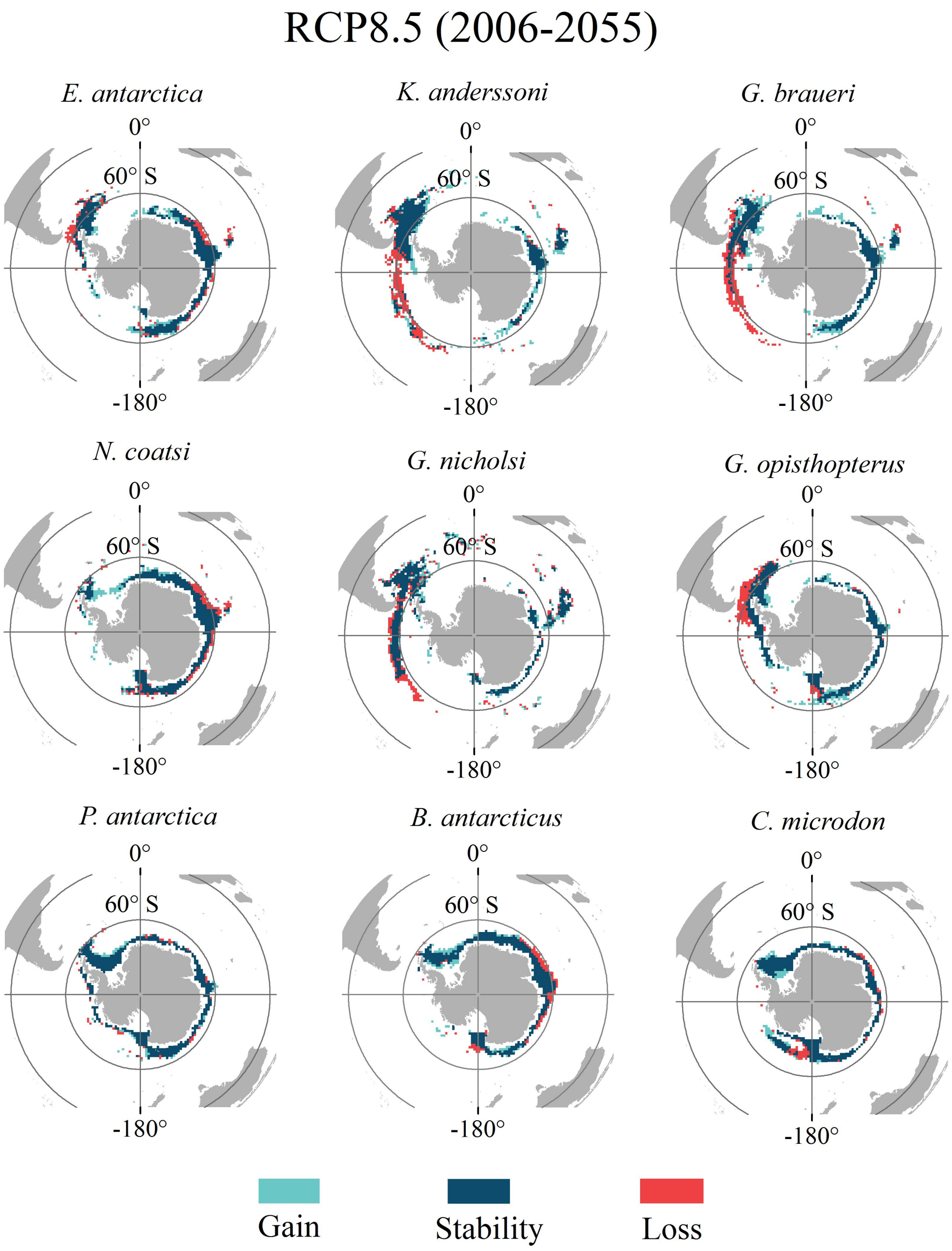

Figure S17. The habitat change in the predicted distribution for nine mesopelagic fish by 2006-2055under medium emission scenario RCP8.5 relative to 1956-2005. The light blue, dark blue, and red areas represent habitat gain (habitat range expansion), habitat stability (no change in habitat), and habitat loss (habitat range contraction), respectively. Representative Concentration Pathway scenarios: RCP.

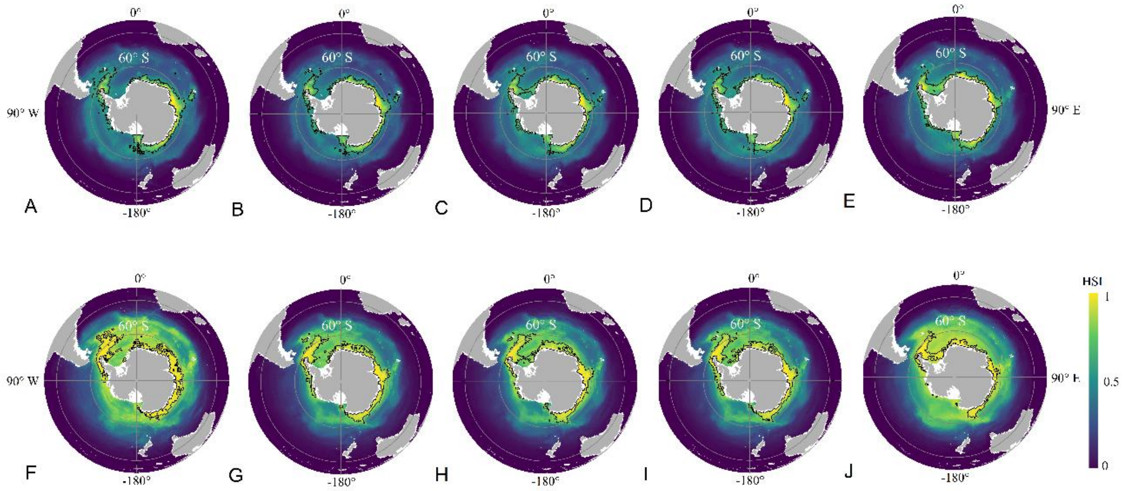

Figure S18. The mean HSI of mesopelagic fish under (**A**) present-day (1956-2005), (**B**) RCP4.5 (2006-2055), (**C**) RCP4.5 (2050-2099), (**D**) RCP8.5 (2006-2055), (**E**) RCP8.5 (2050-2099). The mean HSI of *E. superba* under (**F**) present-day (1956-2005), (**G**) RCP4.5 (2006-2055), (**H**) RCP4.5 (2050-2099), (**I**) RCP8.5 (2006-2055), (**J**) RCP8.5 (2050-2099). The areas within the black line represent important areas (IAs, top 5% HSI). Representative Concentration Pathway scenarios: RCP

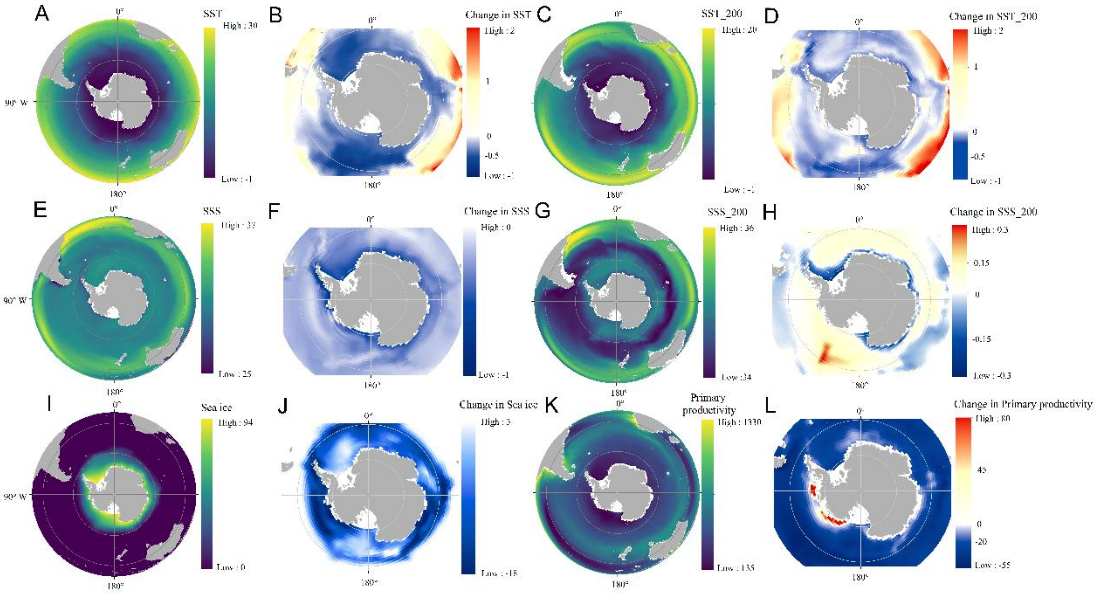

Figure S19. The environmental variables for 1956-2005 in (**A**) SST, (**C**) SST_200, (**E**) SSS, (**G**) SSS_200, (**I**) Sea ice, (**L**) Primary productivity. The change of environmental variables by 2050-2099 under the high emission scenario RCP8.5 relative to 1956-2005 in (**B**) SST, (**D**) SST_200, (**F**) SSS, (**H**) SSS_200, (**J**) Sea ice, (**L**) Primary productivity.

Table S1. Summary of the four Earth System Models (ESMs) used to predict species distribution for three time periods, baseline period (1956-2005), 2006-2055, and 2050-2099.

Model name	Country	Model expansion	Institute
CESM1-BGC	USA	Community Earth System Model, version 1	National Science Foundation, Department of Energy, National Center for Atmospheric Research
GFDL-ESM2G		Geophysical Fluid Dynamics Laboratory Earth System Model with Generalized Ocean Layer Dynamics component	Geophysical Fluid Dynamics Laboratory
GFDL-ESM2M		Geophysical Fluid Dynamics Laboratory Earth System Model with Modular Ocean Model, version 4, component	
HadGEM2-ES	UK	Hadley Centre Global Environment Model, version 2, Earth System	Met Office Hadley Centre (additional HadGEM2-ES realizations contributed by Instituto Nacional de Pesquisas Espaciais)

Table S2. The summary about 28 Earth System Models (ESMs) containing marine environmental predictors, yellow represents that the corresponding ESM contains this environmental predictor. Only four ESMs (CESM1-BGC, GFDL-ESM2G, GFDL-ESM2M and HadGEM2-ES) contain six environmental predictors: sea ice, sea surface salinity (SSS), sea surface temperature (SST), temperature at 200m (T_200), salinity at 200m (S_200) and primary productivity.

RCP4.5	Sea ice	SSS	Primary productivity	SST	S_200	T_200	RCP8.5	Sea ice	SSS	Primary productivity	SST	S_200	T_200
CESM1-BGC							CESM1-BGC						
GFDL-ESM2G							GFDL-ESM2G						
GFDL-ESM2M							GFDL-ESM2M						
HadGEM2-ES							HadGEM2-ES						
IPSL-CM5A-LR							IPSL-CM5A-LR						
IPSL-CM5A-MR							IPSL-CM5A-MR						
IPSL-CM5B-LR							IPSL-CM5B-LR						
CMCC-CESM							CMCC-CESM						

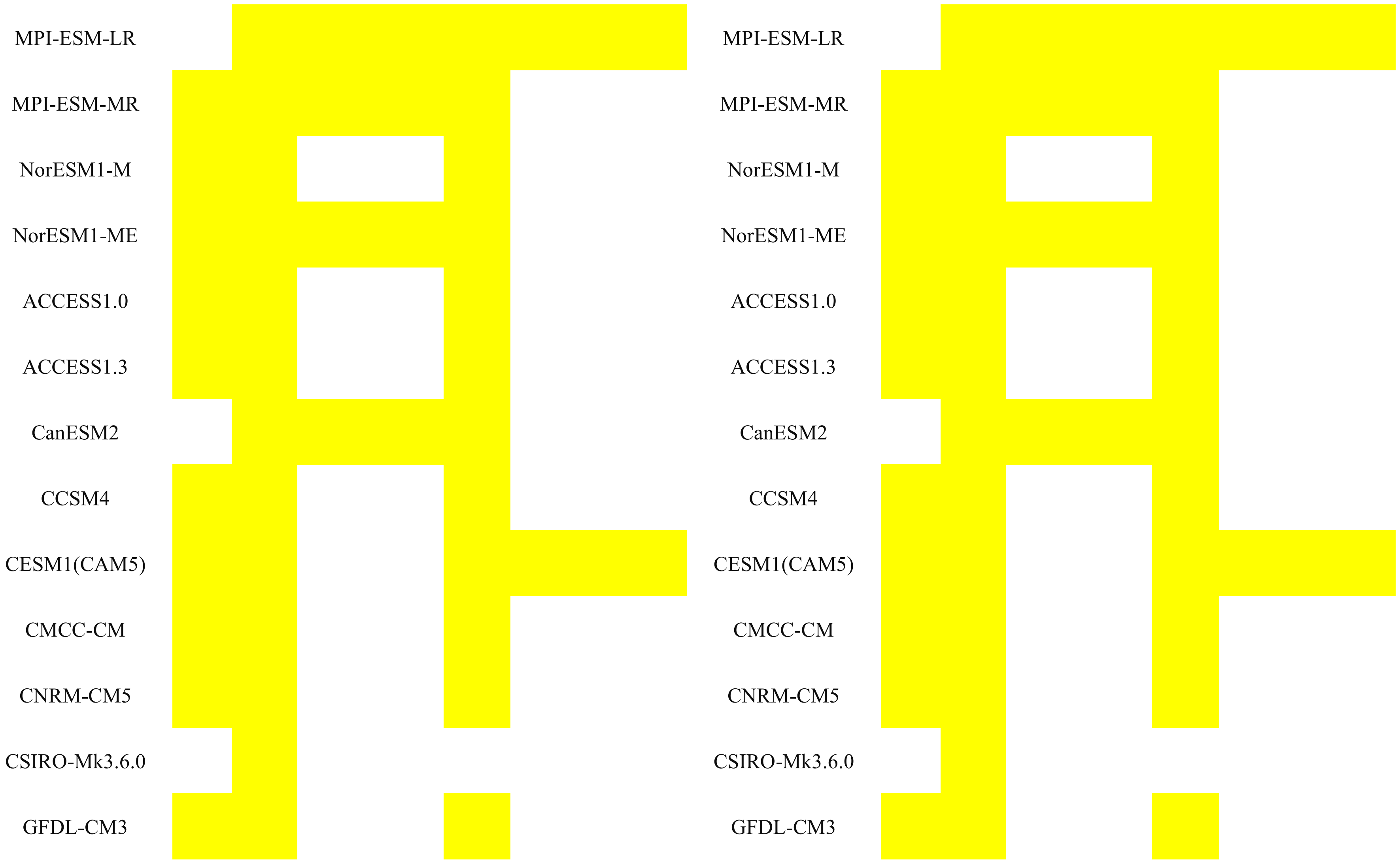
MPI-ESM-LR
MPI-ESM-MR
NorESM1-M
NorESM1-ME
ACCESS1.0
ACCESS1.3
CanESM2
CCSM4
CESM1(CAM5)
CMCC-CM
CNRM-CM5
CSIRO-Mk3.6.0
GFDL-CM3
MPI-ESM-LR
MPI-ESM-MR
NorESM1-M
NorESM1-ME
ACCESS1.0
ACCESS1.3
CanESM2
CCSM4
CESM1(CAM5)
CMCC-CM
CNRM-CM5
CSIRO-Mk3.6.0
GFDL-CM3

GISS-E2-H					GISS-E2-H				
GISS-E2-R					GISS-E2-R				
HadGEM2-AO					HadGEM2-AO				
HadGEM2-CC					HadGEM2-CC				
INM-CM4					INM-CM4				
MIROC5					MIROC5				
MIROC-ESM					MIROC-ESM				
